# MicroRNA Signature and Cellular Characterization of Undifferentiated and Differentiated House Ear Institute-Organ of Corti 1 (HEI-OC1) Cells

**DOI:** 10.1007/s10162-022-00850-6

**Published:** 2022-05-11

**Authors:** Printha Wijesinghe, Desmond A. Nunez, Cathie Garnis

**Affiliations:** 1 Division of Otolaryngology – Head & Neck Surgery, Department of Surgery, Faculty of Medicine, The University of British Columbia, Vancouver, BC V5Z IM9 Canada; 2 Division of Otolaryngology – Head & Neck Surgery, Diamond Health Care Centre, Vancouver General Hospital, 4th Floor-2775 Laurel Street, BC V5Z IM9 Vancouver, Canada; 3 Department of Integrative Oncology, British Columbia Cancer Research Centre, Vancouver, BC V5Z 1L3 Canada

**Keywords:** HEI-OC1 cells, Undifferentiated, Differentiated, miRNA profile, Gene expression

## Abstract

MicroRNAs (miRNAs) regulate gene expressions and control a wide variety of cellular functions. House Ear Institute-Organ of Corti 1 (HEI-OC1) cells are widely used to screen ototoxic drugs and to investigate cellular and genetic alterations in response to various conditions. HEI-OC1 cells are almost exclusively studied under permissive conditions that promote cell replication at the expense of differentiation. Many researchers suggest that permissive culture condition findings are relevant to understanding human hearing disorders. The mature human cochlea however consists of differentiated cells and lacks proliferative capacity. This study therefore aimed to compare the miRNA profiles and cellular characteristics of HEI-OC1 cells cultured under permissive (P-HEI-OC1) and non-permissive (NP-HEI-OC1) conditions. A significant increase in the level of expression of tubulin β1 class VI *(Tubb1)*, e-cadherin *(Cdh1),* espin *(Espn),* and SRY (sex determining region Y)-box2 *(Sox2)* mRNAs was identified in non-permissive cells compared with permissive cells (*P* < 0.05, Kruskal–Wallis H test, 2-sided). miR-200 family, miR-34b/c, and miR-449a/b functionally related cluster miRNAs, rodent-specific maternally imprinted gene *Sfmbt2* intron 10^th^ cluster miRNAs (-466a/ -467a), and miR-17 family were significantly (*P* < 0.05, Welch’s *t*-test, 2-tailed) differentially expressed in non-permissive cells when compared with permissive cells. Putative target genes were significantly predominantly enriched in mitogen-activated protein kinase (MAPK), epidermal growth factor family of receptor tyrosine kinases (ErbB), and Ras signaling pathways in non-permissive cells compared with permissive cells. This distinct miRNA signature of differentiated HEI-OC1 cells could help in understanding miRNA-mediated cellular responses in the adult cochlea.

**Supplementary Information:**

The online version contains supplementary material available at 10.1007/s10162-022-00850-6.

## Introduction

MicroRNAs (miRNAs) are small 18–24-nucleotide non-coding single-stranded RNAs that repress or degrade messenger RNAs (mRNAs) by binding to their complementary sequences at the 3′- untranslated region (3’UTR) (Filipowicz et al. 2008; Obernosterer et al. 2006). miRNAs play critical roles in various kinds of biological processes, such as cellular development, differentiation, metabolism, proliferation, migration, and apoptosis (Pasquinelli 2012; Pelaez and Carthew 2012; Kim et al. 2009), and their altered expression is associated with many human pathologies (Calin and Croce 2006; Esquela-Kerscher and Slack 2006; van Rooij et al. 2006).

MiRNAs play a fundamental role in the regulation of gene expression in the inner ear and associated structures (Mahmoudian-Sani et al. 2017). They are crucial for inner ear development and are involved in the morphogenesis and neurosensory processes that lead to a functional auditory organ (Rudnicki and Avraham 2012). The coordinated expression of miR-183 family members (miR-183, miR-96, and miR-182) has been demonstrated to be particularly important in the development of the sensory cells of the inner ear of mice and other vertebrates (Weston et al. 2006; Sacheli et al. 2009; Li et al. 2010; Friedman et al. 2009). Recent studies show that two single-base mutations in the seed region of miR-96 result in autosomal dominant, progressive hearing loss in both humans and mice (Solda et al. 2012; Mencia et al. 2009; Lewis et al. 2009). This mutation alters the function of miR-96 and their consequent gene expression profile in the mouse organ of Corti such as oncomodulin *(Ocm),* prestin (*Slc26a5*), and growth factor independent 1*(Gfi1)* which have been known to result in deafness and hair cell degeneration (Lewis et al. 2016). These findings demonstrate the importance of miRNA-mediated gene regulation in the cochlea.

House Ear Institute-Organ of Corti 1 (HEI-OC1) cells are one of the few auditory cell lines widely used for research purposes. These cells were derived from the auditory organ of the transgenic mouse Immortomouse™, which harbors a temperature-sensitive mutant of the SV40 large T antigen gene under the control of an interferon-gamma-inducible promoter element (Jat et al. 1991; Kalinec et al. 2003). Incubation of Immortomouse™-derived HEI-OC1 cells at permissive conditions (33 °C, 10 % CO_2_) induces immortalizing gene expression, resulting in de-differentiation of the cells and accelerated proliferation; transferring these cells to non-permissive conditions (39 °C, 5 % CO_2_) results in denaturation of the protein encoded by the gene, leading to decreased proliferation, cell differentiation, and cell death (Kalinec et al. 2003; Devarajan et al. 2002). HEI-OC1 cells are used as an in vitro system for screening of ototoxic drugs and to investigate drug-activated apoptotic pathways, autophagy, senescence, cell protection mechanisms, inflammatory responses, cell differentiation, genetic and epigenetic effects of pharmacological drugs, oxidative and endoplasmic reticulum stress, and other conditions (Kalinec et al. 2016a). Wang et al. (2010) employed a cell model of oxidative stress with HEI-OC1 cells incubated under permissive conditions to determine the impact of oxidative stress on relative miRNA and mRNA transcripts in auditory cells. To the best of our knowledge, no studies have reported the miRNA expression in differentiated HEI-OC1 cells.

Here, we performed experiments to compare the miRNA expression profile of HEI-OC1 cells maintained under non-permissive culture conditions with that of HEI-OC1 cells maintained under permissive conditions. The identity of putative and validated target genes of miRNAs found to be differentially expressed under non-permissive conditions was sought using gene functional analysis. Cellular characterization studies were undertaken to document differences in the morphology, protein, and gene expression of HEI-OC1 cells under permissive and non-permissive conditions.

## Methodology

This study was approved by the Biosafety Committee of the University of British Columbia, Vancouver, Canada.

### Cell Culture

HEI-OC1 cells (kindly provided by Dr. F. Kalinec), derived from the transgenic mouse postnatal organ of Corti, were used to investigate their miRNA expression profiles during proliferation and differentiation. HEI-OC1 cells were cultured under permissive and non-permissive culture conditions as recommended by Kalinec et al. (2016b) to promote proliferation and differentiation, respectively. All cultures were grown in T_25_ flasks (Nunc™ Non-treated) in Dulbecco’s Modified Eagle’s Medium (DMEM), containing 10 % fetal bovine serum (FBS) without supplements and antibiotics in a humidified incubator. Cell morphology was captured with a phase-contrast Zeiss Axio Vert.A1 inverted microscope.

Permissive cultures (P-HEI-OC1 cells) were incubated at 33 °C and 10 % CO_2_ as recommended (Kalinec et al. 2016b). The growth medium was replaced every 2 days. The cells were harvested for experiments once the cultures achieved 100 % confluence usually after 5–7 days of incubation.

Non-permissive cultures were obtained by initially incubating HEI-OC1 cells under permissive conditions until they reach 80–100 % confluence. They were then moved to previously described non-permissive conditions: 39 °C and 5 % CO_2_ to promote cell differentiation (Kalinec et al. 2016b). The cells were maintained over 2 incubation periods: 1 week (NP^1^-) and 2 week (NP^2^-). HEI-OC1 cells under non-permissive culture conditions (NP-HEI-OC1 cells) changed cellular morphologies and started dying as previously described (Kalinec et al. 2003; Devarajan et al. 2002). To minimize the effects of toxins released by dead cells, the growth medium was fully replaced daily and the cultures were harvested after 1-week and 2-week incubation periods, respectively, for further study.

### miRNA Profiling of HEI-OC1 Cells Maintained at Permissive and Non-permissive Conditions

#### miRNA Extraction from HEI-OC1 Cells

Cells were washed with Dulbecco’s phosphate-buffered saline (DPBS) buffer and trypsinized with 0.25 % trypsin–EDTA and incubated at 37 °C for 5 min. Trypsinization was stopped by adding 9 ml of DMEM medium, and the pooled suspension was centrifuged at 1500 rpm for 10 min to obtain the cell pellets for subsequent RNA extractions. miRNA was extracted from the cell pellets using miRNeasy kit (QIAGEN) as per manufacturer’s protocol. Extracted miRNAs were quantified in a BioTek (EPOCH) microplate spectrophotometer using Gen5 software.

#### Reverse Transcription (RT) and Pre-amplification

RT was performed with TaqMan miRNA RT kit (Applied Biosystems) as previously described (Nunez et al. 2020) with slight modifications. Briefly, a RT reaction mixture consisting of 0.8 μl megaplex RT primers (Rodent Pools A + B), 0.2 μl 100 mM dNTPs (with dTTP), 1.5 μl multiscribe reverse transcriptase (50U/μl), 0.8 μl 10X RT buffer, 0.9 μl MgCl2 (25 mM), 0.1 μl RNase inhibitor (20U/μl), 350 ng RNA template, and nuclease-free water to a final volume of 7.5 μl was prepared. RT reaction was carried out on a BioRadT100™ thermal cycler according to the manufacturer’s recommended thermal cycling conditions.

Pre-amplification of the cDNA product after RT was performed using 12.5 µl TaqMan preAmp master mix (2X), 2.5 µl megaplex preAmp primers (Rodent Pools A + B) (10X) and nuclease-free water to a final volume of 25.0 ul in a BioRadT100™ thermal cycler according to the manufacturer’s recommended thermal cycling conditions.

#### TaqMan Low-Density Array (TLDA)

The miRNA profiling of 768 miRNAs was performed with TLDA cards (Rodent Pools A + B Cards Set v3.0). To prepare the real-time PCR reaction mix, 9 µl of diluted pre-amplification product (1:4), 450 µl of TaqMan™ universal PCR master mix (no AmpErase™ UNG) (2X), and 441 µl of nuclease-free water were added to a final volume of 900 µl. One hundred microliters of the PCR reaction mix was loaded onto each row of the 384-well TLDA cards (A or B), centrifuged for 1–2 min at 1200 rpm, sealed carefully and run in a ViiA™ 7 Real-Time PCR System at recommended settings and cycling conditions. HEI-OC1 cells were grown twice under each set of culture conditions (P-, NP^1^-, and NP^2^- HEI-OC1 cells), and TLDA assays were repeated on cells drawn separately from the duplicated cell cultures. Relative miRNA levels were calculated using the comparative threshold cycle (Ct) method (∆∆Ct) normalized to a global mean value and at a cut off Ct level < 35.0.

The TLDA cards tested for 596 *Mus musculus* miRNAs, 78 *Rattus norvegicus* miRNAs, 76 *Homo sapiens* miRNAs, and 18 controls. All non-mouse species’ differentially expressed miRNAs (DEMs) were searched to determine if they shared the same conserved sequences as mouse miRNAs using miRBase database (Release 22.1, http://mirbase.org). Non-mouse DEMs that were homologous to mouse miRNAs were included and non-homologues were excluded from analysis. In addition, DEMs that are not defined as miRNAs currently by the miRBase database (dead entries) were also excluded.

#### Prediction of Putative and Validated Target Genes and Their Functional Enrichment Analysis

The putative and validated target genes of DEMs were obtained using miRWalk3.0 database with filters miRDB and miRTarBase, respectively, at a binding probability of 1.0 within the 3-UTR region (Sticht et al. 2018). The DAVID Bioinformatics Resources 6.8 NIAID/NIH functional annotation tool (da Huang da et al. 2009a; da Huang et al. 2009b) was used to determine if the identified target genes were statistically significantly (at a cut off adjusted *P* value < 0.05) associated with functional terms: Kyoto Encyclopedia of Genes and Genomes (KEGG) and Gene Ontology Biological Process (GOBP).

### Cellular Characterization of Permissive and Non-permissive HEI-OC1 Cells

#### Gene Expression

Eighteen target genes which have been reported in inner ear studies or indicated from our prior miRNA findings in HEI-OC1 cells (Wijesinghe et al. 2021a, b), namely, atonal bHLH transcription factor 1*(Atoh1)* (Hongmiao et al. 2014)*,* POU domain, class 4, transcription factor 3 *(Pou4f3) (*Hertzano et al. 2004)*,* espin *(Espn)* (Zheng et al. 2014)*,* myosin 7a *(Myo7a)* (Hasson et al. 1995, 1997)*,* prestin *(Slc26a5)* (Park et al. 2016)*,* SRY (sex determining region Y)-box2 *(Sox2)* (Kiernan et al. 2005; Hume et al. 2007; Kempfle et al. 2016), paired box 2 *(Pax2)* (Christophorou et al. 2010)*,* cyclin-dependent kinase inhibitor 1b *(Cdkn1b or p27*^*Kip1*^*) (*Chen and Segil 1999)*,* tubulin β1 class VI *(Tubb1)* and tubulin β3 class III *(Tubb3)* (Hallworth and Ludueña 2000; Hallworth et al. 2000; Jensen-Smith et al. 2003), nestin (*Nes*) (Watanabe et al. 2012; Lou et al. 2014), cytokeratin 18 *(Krt18)* (Cyr et al. 2000; Wijesinghe et al. 2021a, b) and vimentin *(Vim)* (Yamasoba and Kondo 2006), scm-like with four mbt domains 2 *(Sfmbt2),* zinc finger E-box-binding homeobox 1 *(Zeb1),* zinc finger E-Box binding homeobox 2 *(Zeb2),* e-cadherin *(Cdh1)*, and tubulin β5 class I *(Tubb5)* were used. Two endogenous controls, glyceraldehyde 3-phosphate dehydrogenase *(Gapdh)* and hypoxanthine guanine phosphoribosyl transferase 1*(Hprt1)* were tested for data normalization. Primer3Plus software (Untergasser et al. 2007) was used to design forward and reverse primers (Table 1).

**Table 1:** Primer sequences used to amplify the target genes in HEI-OC1 cells

**Genes** **(*Mus musculus*)**	**Primers**	**Sequences 5′-3′**
*Atoh1*	Forward	ACATCTCCCAGATCCCACAG
	Reverse	ACAACGATCACCACAGACCA
*Tubb1*	Forward	GCTGCTGTCCATTCAGACAA
	Reverse	GCTCAGAGACCCTGGTGAAG
*Tubb3*	Forward	TGAGGCCTCCTCTCACAAGT
	Reverse	CGCACGACATCTAGGACTGA
*Tubb5*	Forward	TTCAGCTGACCCACTCACTG
	Reverse	AGACAGGGTGGCATTGTAGG
*Cdh1*	Forward	CAAGGACAGCCTTCTTTTCG
	Reverse	TGGACTTCAGCGTCACTTTG
*Espn*	Forward	GCAGAAGATGCAGGAGGAAG
	Reverse	GACTGTTCTTTCGCCCTCTG
*Myo7a*	Forward	CAACATGAAACGCAACAACC
	Reverse	CCAAAGCGGCTAGAGTTGTC
*Nes*	Forward	CCAGAGCTGGACTGGAACTC
	Reverse	ACCTGCCTCTTTTGGTTCCT
*p27*^*Kip1*^	Forward	CAGAATCATAAGCCCCTGGA
	Reverse	TCTGACGAGTCAGGCATTTG
*Pax2*	Forward	TCCCAGTGTCTCATCCATCA
	Reverse	GTTAGAGGCGCTGGAAACAG
*Pou4f3*	Forward	GTCTCAGCGATGTGGAGTCA
	Reverse	TCATGTTGTTGTGCGACAGA
*Slc26a5*	Forward	ACAGTGTGGATGTCGTTGGA
	Reverse	CCATGCTTATTTGCCAAGGT
*Sfmbt2*	Forward	GCATCCTCCAAAAGCAAGAG
	Reverse	GCAGCAGTACTTGGCATTGA
*Sox2*	Forward	AAGGGTTCTTGCTGGGTTTT
	Reverse	AGACCACGAAAACGGTCTTG
*Krt18*	Forward	AGACTTGGTGGTGACAACTGTGG
	Reverse	ATCGAGGCACTCAAGGAAGA
*Vim*	Forward	CGCAGCCTCTATTCCTCATC
	Reverse	GTAGTTGGCAAAGCGGTCAT
*Zeb1*	Forward	GGGGCATCTCACACTTTTGT
	Reverse	AACGGCTGTGAACCAAAAAC
*Zeb2*	Forward	CCACCAGCCCTTTAGGTGTA
	Reverse	CCCTTGTTCTTCTGGCTGAG
*Gapdh*	Forward	CAACAGCAACTCCCACTC
	Reverse	ACCAGGAAATGAGCTTGAC
*Hprt1*	Forward	GCCCCAAAATGGTTAAGGTT
	Reverse	TTGCGCTCATCTTAGGCTTT

RNA (RNeasy^®^ mini kit, QIAGEN) was extracted from P-, NP^1^-, and NP^2^- HEI-OC1 cell pellets which were dissolved in 350 µl RLT buffer containing 0.01 % 14.3 M β-mercaptoethanol according to the manufacturer’s protocol. The quantity and quality of extracted RNA were determined prior to cDNA preparation. cDNA synthesis was performed with SuperScript™ VILO™ cDNA synthesis kit (Invitrogen) as per manufacturer’s protocol in a BioRadT100™ thermal cycler. Synthesized cDNAs were then diluted to a concentration of 5 ng/µl.

In brief, the RT-qPCR reaction mix per well consisted of 1 µl of HyPure™ molecular biology grade water, 5 µl SYBR select master mix at the manufacturer’s supplied concentration, 1 µl of each forward and reverse primers (10 µM), and 2 µl of diluted cDNA (5 ng/µl). After the reaction mix was added to the wells, the plate was centrifuged for a few seconds in a Mini PCR Plate Spinner. RT-qPCR with an initial denaturing step of 95 °C for 10 min and followed by 40 amplification cycles of 15 s at 95 °C and 1 min at 60 °C duration was undertaken on a Quant Studio™ 3 Real-Time PCR system (Applied Biosystems). All target gene tests were repeated a minimum of three times on each sample. Relative mRNA levels were determined using the comparative cycle threshold method at a cut-off Ct < 40.0. *Gapdh* was used as a reference gene for data normalization. The relative mRNA level was expressed as the mRNA copies of the gene of interest per 1000 copies of *Gapdh* mRNA [2^−∆Ct^/1000 = 1000/2^∆Ct^ = 1000/2^^(avg. target gene Ct – avg. reference gene Ct)^] (Schmittgen and Livak 2008; Huang et al. 2014).

#### mRNA-miRNA Interactions

Since the tested target genes were primarily selected from previous inner ear studies, it is worthwhile to predict their biological target miRNAs to determine the mRNA-miRNA interactions. Therefore, target genes used for the cellular characterization of P-, NP^1^-, and NP^2^- HEI-OC1 cells were then searched for their biological target miRNAs by searching for the presence of conserved sequences (8mer and 7mer) that match the seed region of each miRNA with TargetScanMouse version 7.2 (Agarwal et al. 2015). miRNAs that shared poorly conserved sequences were excluded. mRNA-miRNA interactions are illustrated using Cytoscape version 3.7.1 (Shannon et al. 2003).

#### Fluorescence Immunocytochemistry (ICC)

HEI-OC1 cells were grown in 8-well chamber slides (Lab Tek, Permanox TC Surface, Treated) under varying culture conditions as described above. The immunocytochemical characteristics of the HEI-OC1 cells under permissive and non-permissive culture conditions were determined using 6 selected protein markers, namely, inner (myosin 7a) and outer (prestin) hair cell markers, stem/progenitor cell markers Sox2 and nestin, and epithelial-mesenchymal transition markers (EMT) e-cadherin and vimentin.

HEI-OC1 cells that reached ≥ 80 % confluence were used for fluorescence ICC. Culture medium was removed, and the cells were washed for 1 min in DPBS 3 times. The cells were then fixed in 4 % paraformaldehyde for 15 min, followed by permeabilization in 0.1 % Triton-X 100 for 15 min. These cells were washed for 1 min in DPBS, 3 times. Thereafter, the cells were blocked using 3 % bovine serum albumin (BSA) at room temperature for 30 min prior to incubation at 4 °C overnight with primary antibodies [myosin 7a 1:100 dilution (rabbit polyclonal- ab3481, ABCAM); prestin 1:100 dilution (goat polyclonal- SC22692, Santa Cruz Biotechnology); nestin, neural stem cell marker 1:100 dilution (rabbit polyclonal- ab92391, ABCAM); Sox2 1:100 dilution (rabbit polyclonal- ab97959 ABCAM); e-cadherin 1:100 (rabbit polyclonal- ab15148), and vimentin 1:200 dilution (rabbit polyclonal- PA5-27,231, Invitrogen)] dissolved in 3 % BSA. The following day, primary antibodies were drained, and the chamber slides washed for 1 min in DPBS 3 times. Then, the cells were incubated at room temperature with secondary antibodies in the dark [donkey anti-rabbit Alexa Fluor^®^488 1:500 dilution (A21206, Invitrogen) and donkey anti-goat Alexa Fluor^®^488 1:500 dilution (A11055, Invitrogen)], respectively, to the primary antibodies for 1 h in a shaker. After incubation, secondary antibodies were drained, and the chamber slides washed for 1 min in DPBS 3 times. The cells were then mounted with ProLong™ Gold Antifade Mountant with DAPI (P36931, Invitrogen). Images were captured using a Zeiss Axio Vert.A1 Inverted Microscope. Immunofluorescence staining was performed in duplicate for each P-, NP^1^-, and NP^2^- HEI-OC1 cell cultures, respectively.

The number of immunofluorescence-positive cells per 400 × magnification field was recorded to determine the protein expressions semi quantitatively. Viable (DAPI positive nuclei) cells were relatively sparse and unevenly distributed under non-permissive culture conditions. Thus, areas in each culture with high numbers of DAPI-positive nuclei were selected for counting the number of antibody positively and negatively stained cells. P-HEI-OC1 cultures contained high levels of evenly distributed viable cells making selection of ideal high-power fields for study straightforward. Counts from 5 non-overlapping fields were recorded in all culture conditions and the average counts determined.

#### Statistical Analysis

Normalized mean Ct values of miRNAs expressed in P-, NP^1^-, and NP^2^- HEI-OC1 cell cultures (NP^1^ vs. P-, NP^2^- vs. P-, and NP^2^ vs. NP^1^-) were statistically compared using Welch’s *t*-test (2-tailed). *P* values of all tested miRNAs were subjected to Benjamini–Hochberg correction (false discovery rate, 50 %) at a significance level of *P* < 0.05 (McDonald 2014; Benjamini and Hochberg 1995) for each inter-group comparison. DEMs were then defined as those that demonstrated a statistically significant intergroup fold difference (2^−∆∆Ct^) > 2.0 (upregulated) and/ or < 0.5 (downregulated).

For the gene expression, normalized mean Ct values and for protein expression, the proportions of antibody positively stained cells were compared across P-, NP^1^-, and NP^2^-HEI-OC1 cell cultures using non-parametric Kruskal–Wallis *H* test (2-sided), followed by Dunn’s post hoc test at a Bonferroni-adjusted significance level of *P* < 0.05 for multiple tests.

Welch’s *t*-test for miRNA expression and non-parametric Kruskal–Wallis *H* test for gene and protein expression were applied as the standard deviations were different for some tested miRNAs, genes, and proteins in permissive and non-permissive cultures. SPSS version 25.0 (IBM Corp., Armonk, New York) and GraphPad Prism 8 (GraphPad Software Inc., San Diego, CA) were used for statistical analysis and to generate graphs.

## Results

### Cell Culture Morphology

**Fig. 1:**
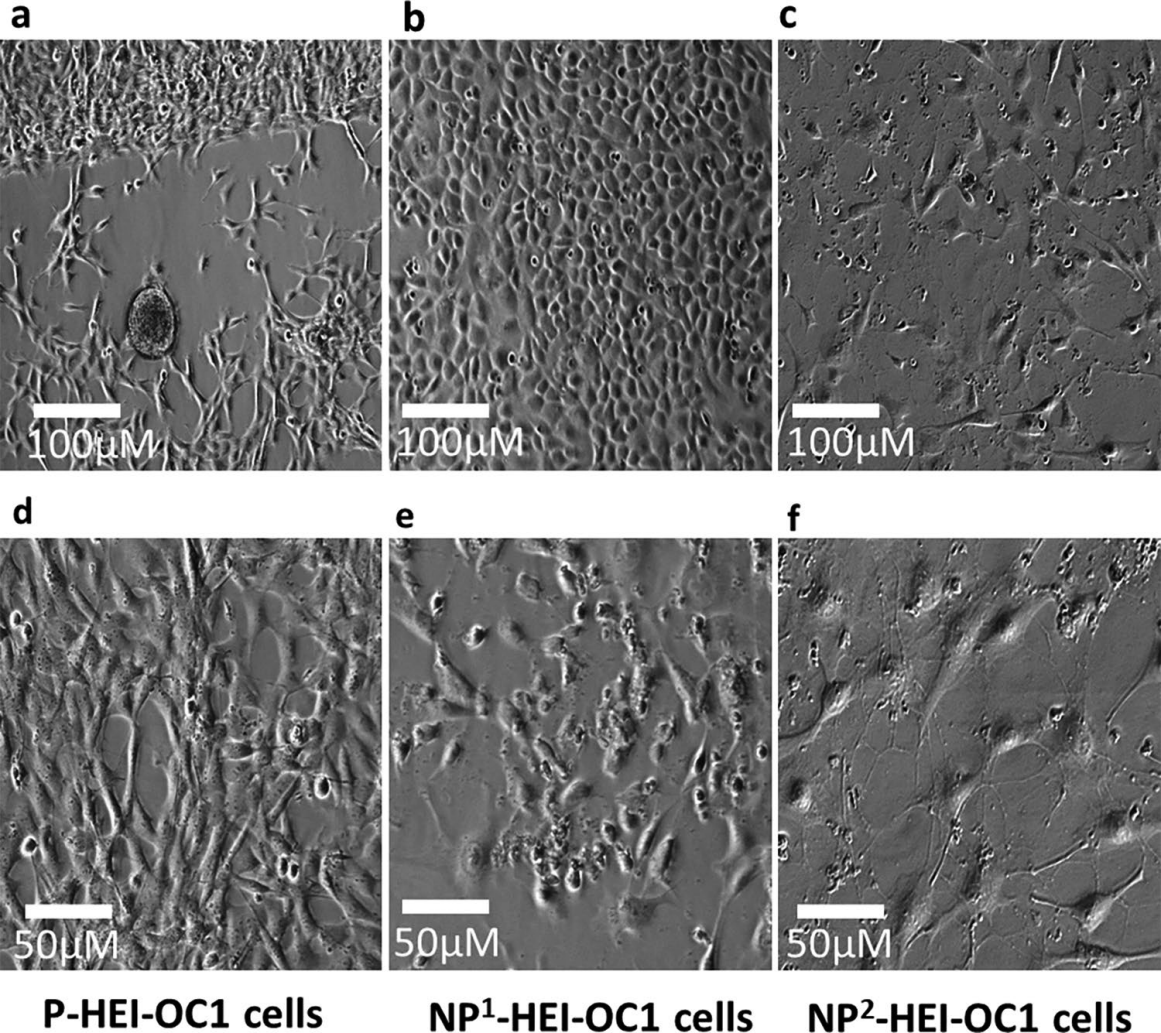
Cell culture morphologies of HEI-OC1 cells maintained at permissive and non-permissive culture conditions. **a** and **d** P-HEI-OC1 cells at 5th and 7th (> 80 % confluence) day of incubation, respectively. **b** and **e** NP^1^-HE1-OC1 cells at 9th and 14th day of incubation, respectively. **c** and **f** NP^2^-HE1-OC1 cells at 16th and 21st day of incubation, respectively. Images were captured with phase-contrast microscopy (scale bars are indicated)

Culturing HEI-OC1 cells under permissive conditions facilitated proliferation, while non-permissive conditions promoted differentiation. Permissive condition cultures demonstrated small cell size and increased cell numbers (Fig. 1a and d), in keeping with a high proliferative phase. The morphology changed from spindle-shaped to cobblestone-shaped cells when the cells were transitioned from permissive to non-permissive conditions as illustrated (Fig. 1d and b, respectively). The number of cells decreased, individual cell size increased, and nuclear clumping and debris accumulation increased consistent with more cell death at 2 weeks’ incubation under non-permissive conditions (Fig. 1e, c, and f).

### DEMs in HEI-OC1 Cells Maintained at Permissive and Non-permissive Culture Conditions

**Fig. 2:**
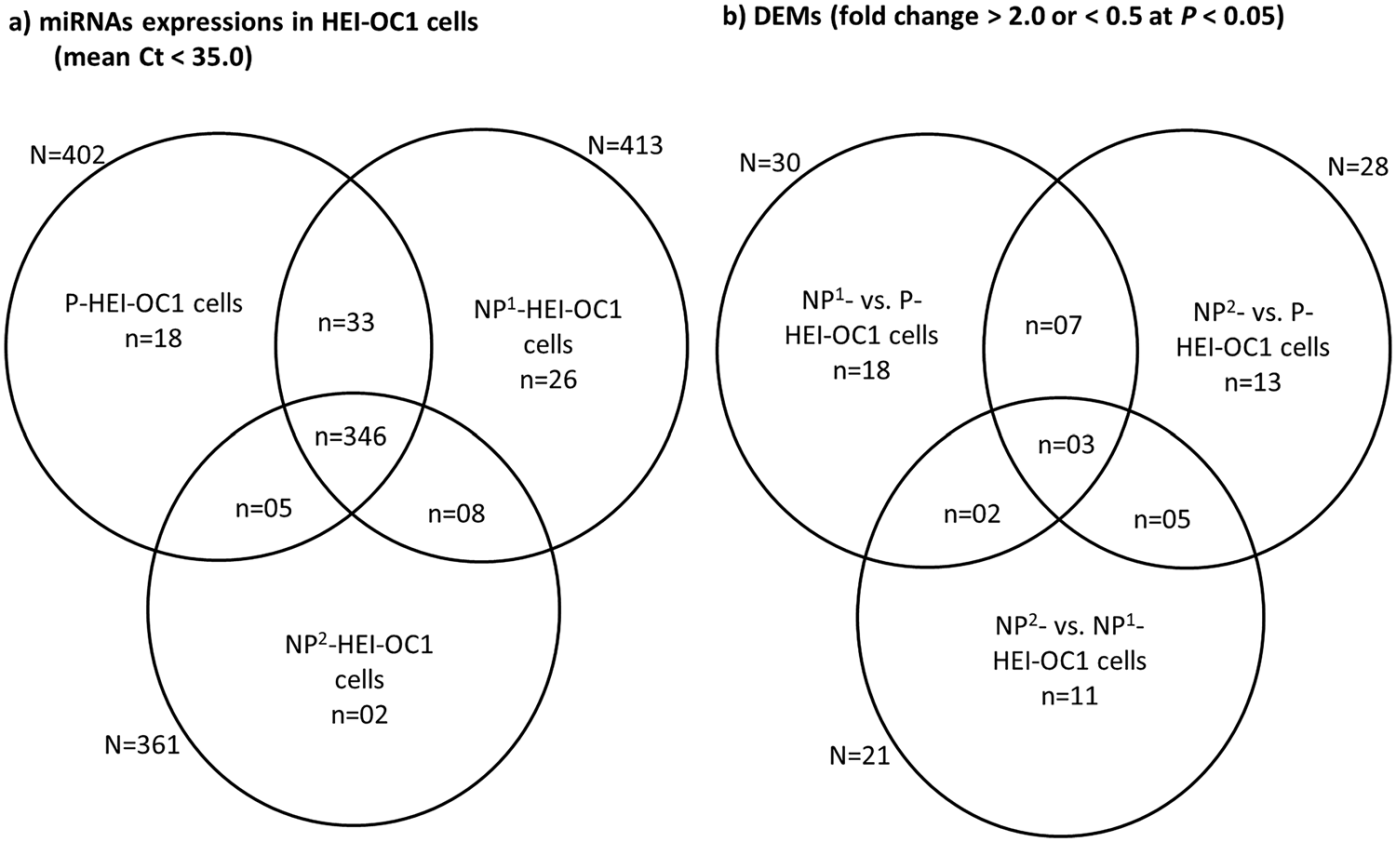
miRNA expressions in permissive and non-permissive HEI-OC1 cells. **a** miRNAs that were expressed at a mean Ct < 35 in P-, NP^1^, and NP^2^-HEI-OC1 cells. **b** DEMs among P-, NP^1^, and NP^2^-HEI-OC1 cells (as per miRBase, dead entries, and the miRNAs which are a non-mouse species and non-homologue to mouse miRNAs are excluded from Fig. 2b)

In P-HEI-OC1 cells, 402 out of 768 miRNAs tested were expressed at a mean Ct cut off level < 35.0 (Fig. 2a). Similarly, 413 and 361 miRNAs were expressed in NP^1^- and NP^2^- HEI-OC1 cells, respectively (Fig. 2a). In all three cultures, 346 miRNAs were detected (Suppl. Tables 1 and 2).

Thirty, 28, and 21 DEMs were identified in NP^1^- versus (vs) P-, NP^2^- vs. P-, and NP^2^- vs. NP^1^- HEI-OC1 cells, respectively (Fig. 2b). All up- and downregulated DEMs are summarized (Tables 2, 3, and 4, respectively). Three miRNAs miR-1948-3p, -20a-3p, and -486a-5p were differentially expressed in all three comparisons, and among them, miR-20a-3p was commonly downregulated. Seven miRNAs hsa-miR-200c-3p, hsa-miR-214-3p, -miR-186-3p, -1971, -2134, -222-3p, and -34c-3p were consistently differentially expressed in non-permissive cells compared with permissive cells (NP^1^- and NP^2^- vs. P-HEI-OC1 cells). miRNAs hsa-200c-3p, hsa-miR-214-3p, -miR-222-3p, and -34c-3p were upregulated and miR-186-3p, -1971 and -2134 were downregulated. Two DEMs miR-449a-5p and -301b-3p were upregulated in NP^1^-HEI-OC1 cells compared with either P- or NP^2^- HEI-OC1 cells. Five DEMs hsa-miR-33a-3p, hsa-miR-340-5p, -miR-199a-5p, -24–2-5p, and -340-3p were all downregulated in NP^2^-HEI-OC1 cells compared with either P- or NP^1^- HEI-OC1 cells. miR-340 (-3p/-5p) was consistently downregulated in NP^2^-HEI-OC1 cells. Rodent-specific maternally imprinted gene *Sfmbt2* intron 10^th^ cluster miRNAs miR-466a-3p and -467a-5p were downregulated in NP^1^- and NP^2^- HEI-OC1 cells, respectively, compared with P-HEI-OC1 cells.

**Table 2:** Significantly differentially expressed miRNAs in NP^1^-HEI-OC1 cells compared with P-HEI-OC1 cells

DEMs upregulated (*n* = 21)	FC (> 2.0)	*P*^*a*^ value (< 0.05)	DEMs downregulated (*n* = 09)	FC (< 0.5)	*P*^*a*^ value (< 0.05)
miR-146b-5p	10.67	0.0005	miR-2134	0.03	0.0032
hsa-miR-93-3p	3.66	0.0006	miR-466 k	0.19	0.0133
miR-872-3p	4.97	0.0032	miR-20a-3p	0.23	0.0135
miR-200c-3p	14.06	0.0033	miR-712-5p	0.03	0.0194
miR-877-3p	14.05	0.0041	miR-466a-3p	0.16	0.0238
miR-133a-3p	19.24	0.0052	miR-1971	0.11	0.0257
miR-1948-3p	17.89	0.0057	miR-1903	0.04	0.0267
miR-339-3p	4.20	0.0074	miR-701-5p	0.15	0.0288
miR-574-3p	13.07	0.011	miR-186-3p	0.24	0.0292
miR-301b-3p	3.33	0.0123			
miR-1932	13.47	0.0124			
miR-129–2-3p	4.80	0.0156			
miR-146a-5p	11.48	0.016			
miR-34c-3p	65.36	0.0176			
hsa-miR-200c-3p	51.03	0.0191			
hsa-miR-214-3p	7.63	0.0192			
miR-199a-3p	3.65	0.0235			
miR-222-3p	9.46	0.025			
miR-486a-5p	3.74	0.0256			
miR-449a-5p	9.23	0.0298			
miR-547-3p	8.75	0.0307

**Table 3:** Significantly differentially expressed miRNAs in NP^2^-HEI-OC1 cells compared with P-HEI-OC1 cells

DEMs upregulated (*n* = 10)	FC (> 2.0)	*P*^*a*^ value (< 0.05)	DEMs downregulated (*n* = 18)	FC (< 0.5)	*P*^*a*^ value (< 0.05)
miR-1948-3p	4.25	0.009	miR-20a-3p	0.07	0.0041
miR-449b	4.84	0.0094	hsa-miR-340-5p	0.07	0.0074
hsa-miR-425-5p	16.22	0.0117	miR-486a-5p	0.14	0.0078
hsa-miR-214-3p	6.42	0.0121	miR-2134	0.06	0.0096
rno-miR-664-3p	32.20	0.0202	miR-340-3p	0.05	0.0204
miR-34c-3p	17.97	0.0203	miR-1971	0.09	0.0213
hsa-miR-200c-3p	44.58	0.021	hsa-miR-33a-3p	0.05	0.0213
miR-674-3p	3.51	0.0363	miR-467a-5p	0.03	0.0237
miR-222-3p	4.15	0.0449	hsa-miR-196a-5p	0.29	0.0269
miR-1943-5p	2.96	0.0476	miR-24–2-5p	0.42	0.027
			miR-322-3p	0.17	0.0274
			miR-199a-5p	0.20	0.0304
			miR-322-5p	0.11	0.0307
			hsa-miR-106b-3p	0.29	0.0369
			miR-17-5p	0.13	0.041
			miR-186-3p	0.03	0.0412
			miR-340-5p	0.03	0.0431
			miR-99a-5p	0.11	0.0434

**Table 4:** Significantly differentially expressed miRNAs in NP^2^-HEI-OC1 cells compared with NP^1^-HEI-OC1 cells

DEMs downregulated (*n* = 21)	FC (< 0.5)	*P*^*a*^ value (< 0.05)
miR-34b-5p	0.11	0.0013
miR-1948-3p	0.24	0.0028
miR-449a-5p	0.06	0.0066
miR-24–2-5p	0.42	0.007
miR-140-5p	0.08	0.008
miR-130a-3p	0.25	0.0092
hsa-miR-744-3p	0.33	0.0101
let-7d-3p	0.05	0.015
hsa-miR-340-5p	0.13	0.0157
miR-20a-3p	0.30	0.0162
miR-486a-5p	0.04	0.0169
miR-340-3p	0.05	0.0208
miR-301a-3p	0.18	0.022
miR-301b-3p	0.15	0.0236
hsa-miR-140-3p	0.10	0.0266
hsa-miR-33a-3p	0.18	0.0318
miR-345-5p	0.49	0.0341
miR-106a-5p	0.33	0.0354
miR-199a-5p	0.20	0.0402
miR-484	0.20	0.042
hsa-miR-423-3p	0.49	0.0433

DEMs with twofold intergroup difference at* P* < 0.05; FDR corrected by Benjamini–Hochberg procedure are presented. Non-mouse species DEMs (rno-miR-350/ -146b-5p/ and hsa-miR-l48b-5p) which were not homologues of mouse miRNAs are excluded (FC, fold change; *P*^*a*^, actual *P* value).

DEMs with twofold intergroup difference at* P* < 0.05; FDR corrected by Benjamini–Hochberg procedure are presented. Non-mouse species DEMs (rno-miR-350/ -99a-3p/ and -l48b-5p) which were not homologues of mouse miRNAs are excluded (FC, fold change; *P*^*a*^, actual *P* value).

DEMs with twofold intergroup difference at *P* < 0.05; FDR corrected by Benjamini–Hochberg procedure are presented. Non-mouse species DEMs (rno-miR-99a-3p/ and -350) which were not homologues of mouse miRNAs are excluded (FC, fold change; *P*^*a*^, actual *P* value).

### Putative Target Genes’ Enriched Significant Functional Annotations for DEMs in Non-permissive HEI-OC1 Cells

**Fig. 3:**
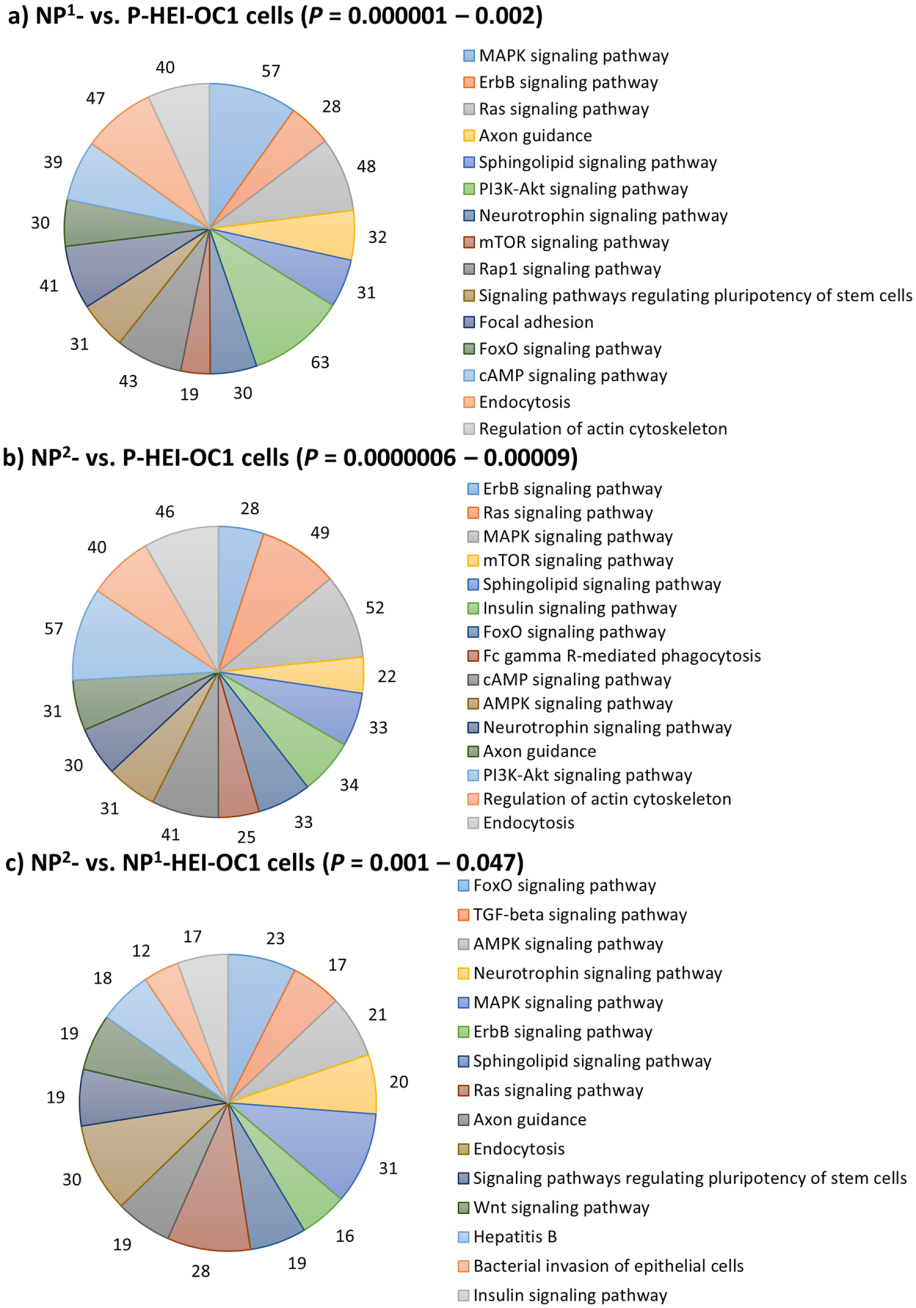
Significantly enriched KEGG pathways for putative target genes of DEMs in non-permissive HEI-OC1 cells. **a** NP^1^- compared with P-HEI-OC1 cells. **b** NP^2^- compared with P-HEI-OC1 cells. **c** NP^2^- compared with NP^1^-HEI-OC1 cells. Significantly enriched top 15 KEGG pathways are presented excluding cancers. The number of genes involved in each pathway is labelled in each panel and the legend keys are presented at descending corrected *P* values (cut-off *P* < 0.05)

**Fig. 4:**
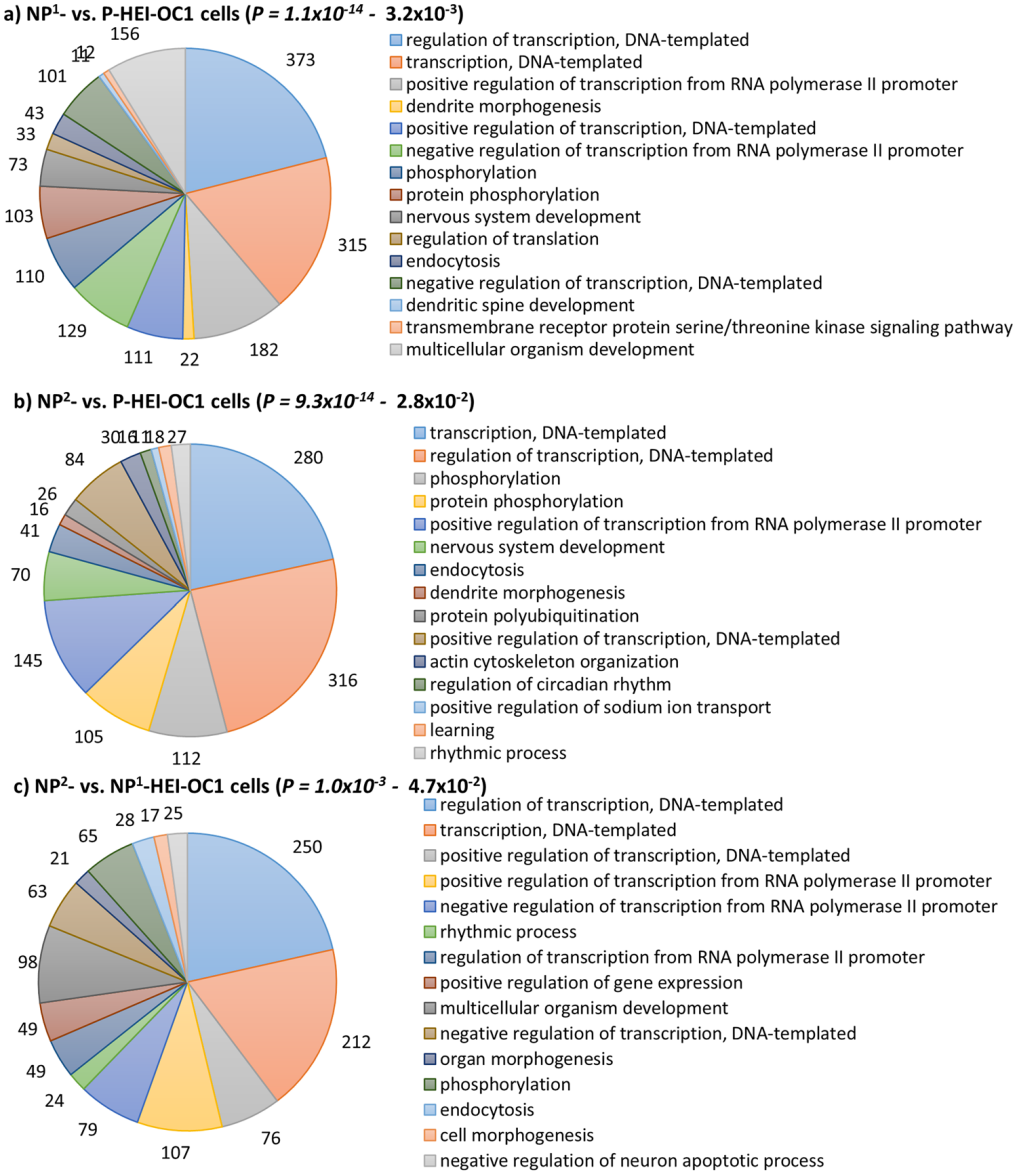
Significantly enriched GOBP terms of putative target genes of DEMs in non-permissive HEI-OC1 cells. **a** NP^1^- compared with P-HEI-OC1 cells. **b** NP^2^- compared with P-HEI-OC1 cells. **c** NP^2^- compared with NP^1^-HEI-OC1 cells. Significantly enriched top 15 GOBP terms are presented. The number of genes involved in each pathway is labelled in each panel and the legend keys are presented at descending corrected *P* values (cut-off *P* < 0.05)

There were 2211, 1876, and 1232 putative target genes identified for DEMs in NP^1^- compared with P-HEI-OC1 cells, NP^2^- compared with P-HEI-OC1 cells, and NP^2^- compared with NP^1^-HEI-OC1 cells, respectively. Of these putative genes, 786, 674, and 431 were recognized by KEGG pathways (Fig. 3), and 1955, 1661, and 1086 of these genes by GOBP terms (Fig. 4), respectively. Excluding cancers, mitogen-activated protein kinase (MAPK), epidermal growth factor family of receptor tyrosine kinases (ErbB), and Ras were predominantly enriched KEGG pathways in non-permissive cells when compared with permissive cells (*P* < 0.0001) (Fig. 3a and b, respectively). The forkhead box O (FoxO), transforming growth factor beta (TGF-beta), and AMPK (5′ adenosine monophosphate-activated protein kinase) were predominantly enriched KEGG pathways in NP^2^-HEI-OC1 cells when compared with NP^1^-HEI-OC1 cells (*P* < 0.01) (Fig. 3c). Excluding transcription and regulation of transcription, the GOBP terms dendrite morphogenesis, phosphorylation, protein phosphorylation, and nervous system development were predominantly enriched in non-permissive cells when compared with permissive cells (*P* < 0.01) (Fig. 4a and b, respectively). GOBP terms: rhythmic process, multicellular organism development, and organ morphogenesis were predominantly enriched in NP^2^-HEI-OC1 cells when compared with NP^1^-HEI-OC1 cells (*P* < 0.00001) (Fig. 4c).

### Validated Target Genes’ Enriched Significant Functional Annotations for DEMs in Non-permissive HEI-OC1 Cells

**Fig. 5:**
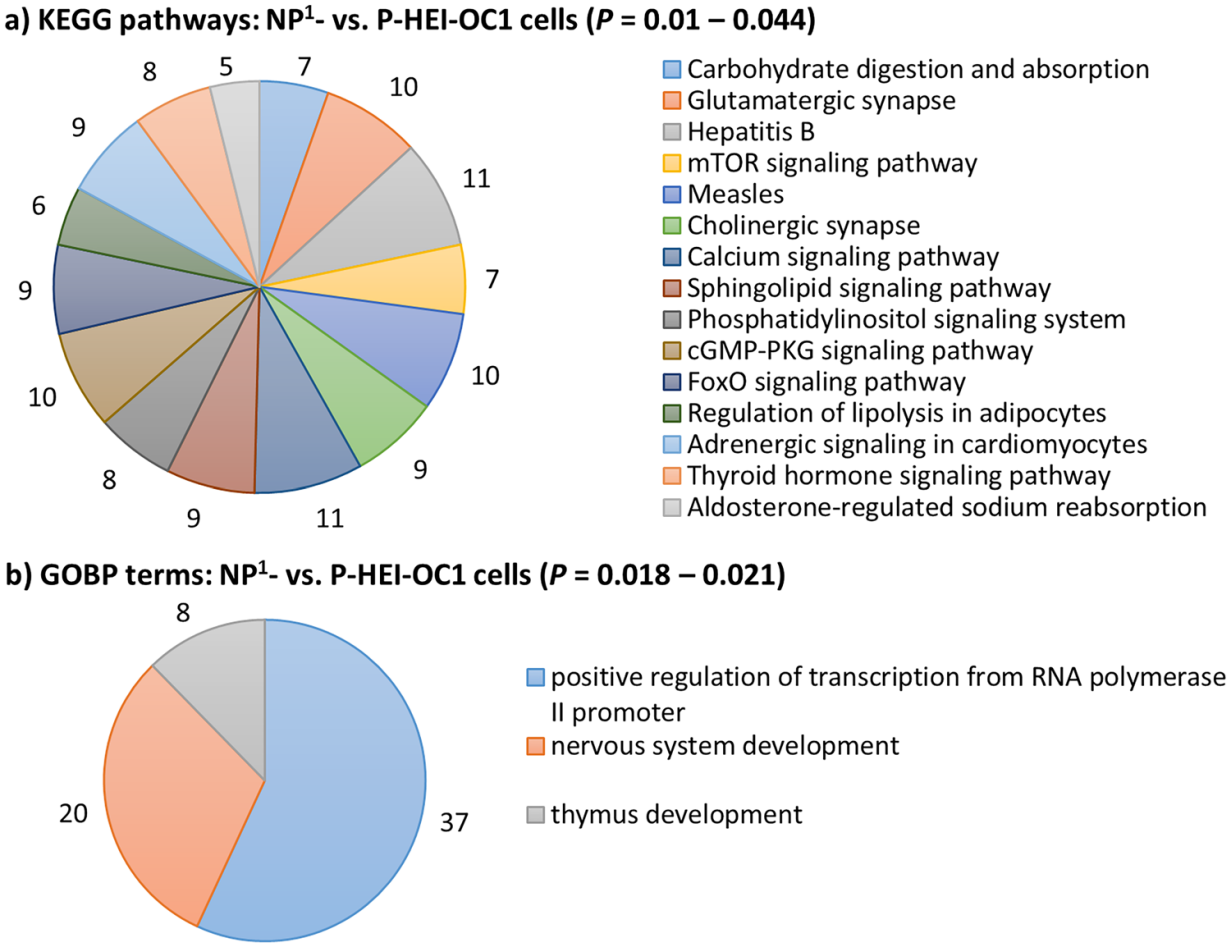
Significantly enriched functional annotations for validated target genes of DEMs in non-permissive HEI-OC1 cells. **a** KEGG pathways in NP^1^- compared with P-HEI-OC1 cells. **b** GOBP terms in NP^1^- compared with P-HEI-OC1 cells. The number of genes involved in each pathway is labelled in each panel, and the legend keys are presented at descending corrected *P* values (cut off *P* < 0.05)

There were 333, 207, and 125 validated target genes identified for DEMs in NP^1^- vs. P-, NP^2^- vs. P-, and NP^2^- vs. NP^1^- HEI-OC1 cells, respectively. Of these validated genes, 134, 80, and 45 were recognized by KEGG pathways, and 301, 185, and 112 of these genes by GOBP terms, respectively (Fig. 5). Carbohydrate digestion and absorption, glutamatergic synapse, and mechanistic or mammalian target of rapamycin (mTOR) signaling were predominantly enriched KEGG pathways in NP^1^-HEI-OC1 cells when compared with P-HEI-OC1 cells (*P* < 0.001) (Fig. 5a). Signaling pathways regulating pluripotency of stem cells was the only significantly enriched KEGG pathway in NP^2^-HEI-OC1 cells when compared with P-HEI-OC1 cells (*P* = 0.01). Excluding transcription and regulation of transcription, the GOBP term nervous system development was predominantly enriched in NP^1^-HEI-OC1 cells when compared with P-HEI-OC1 cells (*P* < 0.05) (Fig. 5b). None of the KEGG pathways and GOBP terms was significantly enriched for validated target gens of DEMs in NP^2^-HEI-OC1 cells when compared with NP^1^-HEI-OC1 cells (*P* > 0.05).

### Relative Gene Expressions in Permissive and Non-permissive HEI-OC1 Cells

**Fig. 6:**
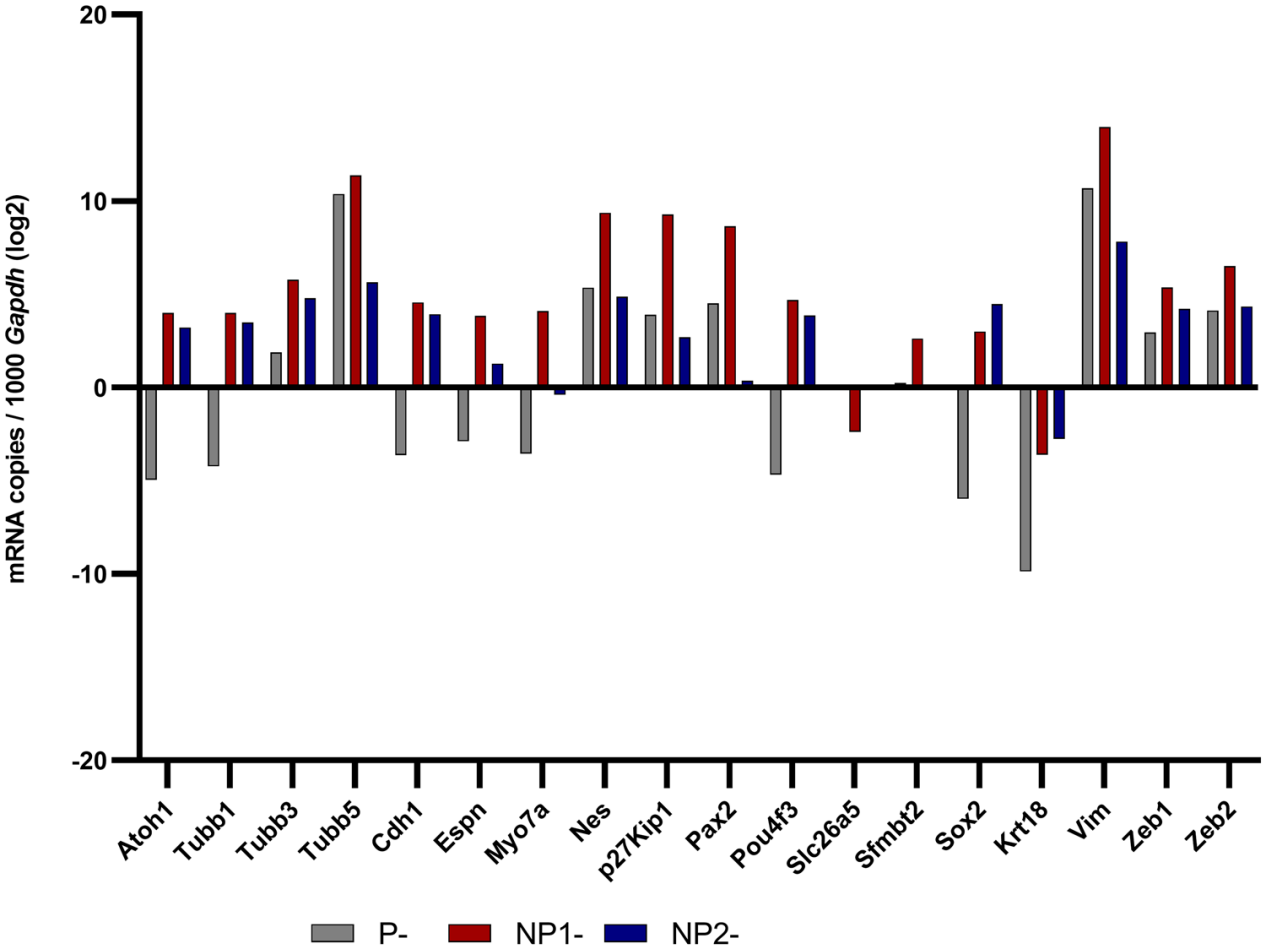
Relative mRNA levels of target genes expressed in P-, NP^1^-, NP^2^-HEI-OC1 cells. Genes encoding for *Atoh1, Tubb1, Tubb3, Tubb5, Cdh1, Espn, Myo7a, Nes, p27*^*Kip1*^*, Pax2, Pou4f3*, *Slc26a5, Sfmbt2, Sox2, Krt18, Vim, Zeb1, and Zeb2* are presented using mRNA copies gene of interest per 1000 *Gadph* mRNA in log2. Bar graph was generated with GraphPad Prism 8 (GraphPad Software Inc., San Diego, CA)

**Fig. 7:**
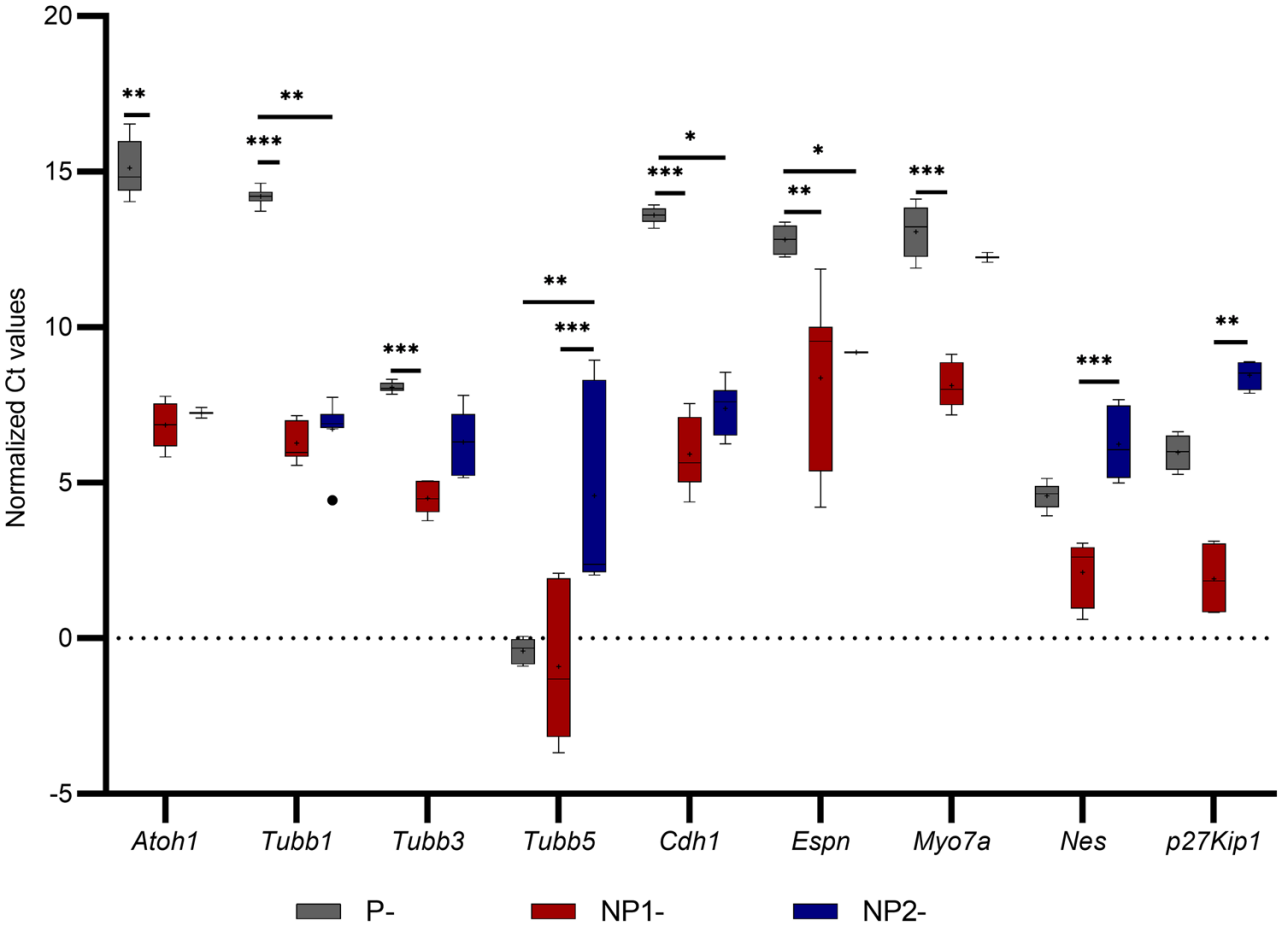
A Box and Whisker plot to illustrate the normalized Ct values (< 40) obtained for target genes *Atoh1, Tubb1, Tubb3, Tubb5, Cdh1, Espn, Myo7a, Nes* and *p27*^*Kip1*^ in P-, NP^1^-, NP^2^-HEI-OC1 cells. Box and Whisker plot was generated with GraphPad Prism 8 (GraphPad Software Inc., San Diego, CA). Whiskers were made using Tukey’s method (whiskers extend to 1.5 × IQR). Ct values normalized to endogenous control *Gapdh* were compared with Welch’s *t*-test (2-tailed) and the FDR was corrected by Benjamini–Hochberg procedure. Normalized mean Ct values were compared using non-parametric Kruskal–Wallis H test (2-sided) followed by Dunn’s post hoc test. Significantly different inter-group differences in normalized mean Ct values expressed at a Bonferroni adjusted for multiple comparisons significance level of *P* < 0.05 indicated as < 0.05*, < 0.01**, and < 0.001***

**Fig. 8:**
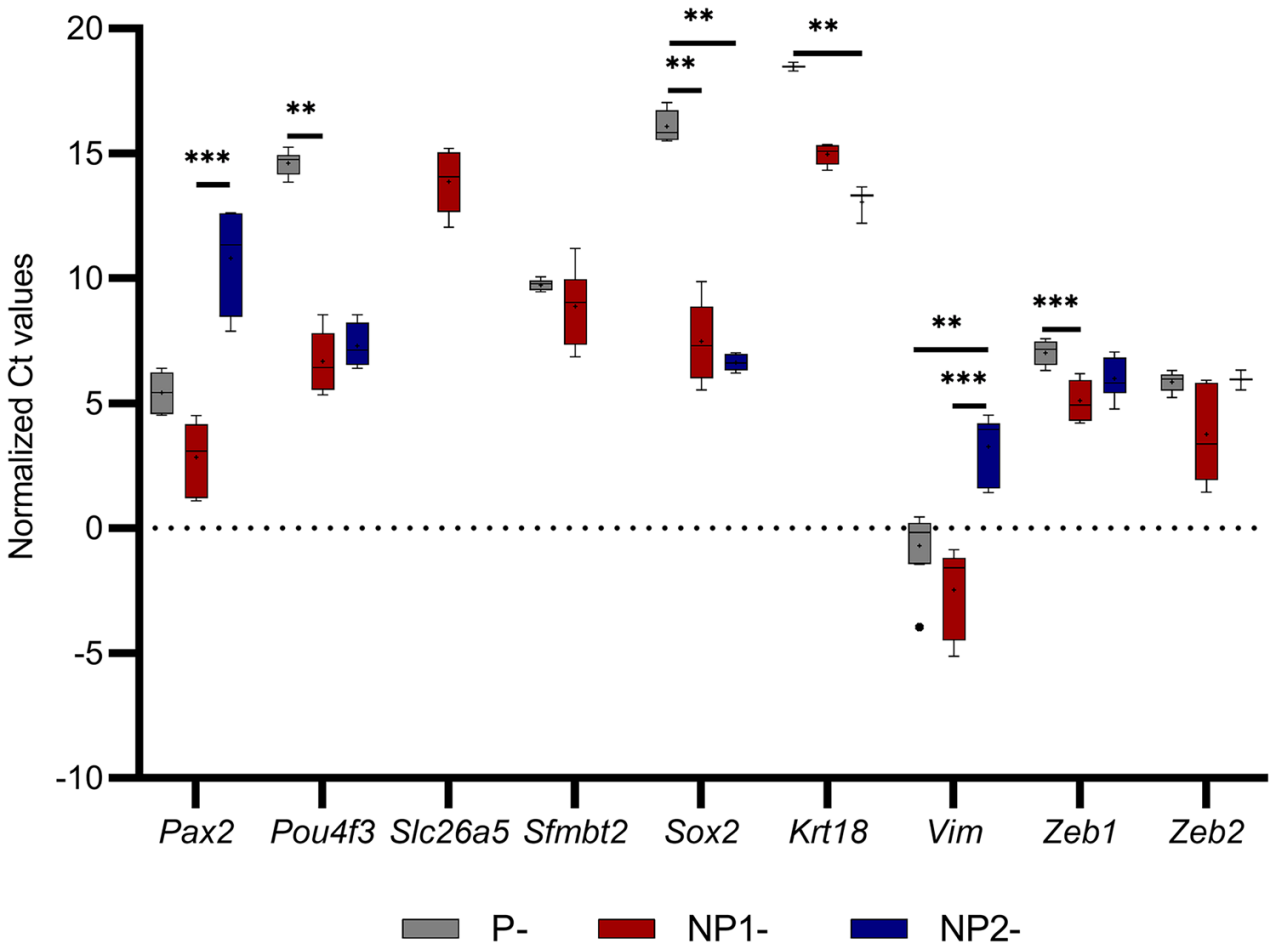
A Box and Whisker plot to illustrate the normalized Ct values (< 40) obtained for target genes *Pax2, Pou4f3*, *Slc26a5, Sfmbt2, Sox2, Krt18, Vim, Zeb1,* and *Zeb2* in P-, NP^1^-, NP^2^-HEI-OC1 cells. Box and Whisker plot was generated with GraphPad Prism 8 (GraphPad Software Inc., San Diego, CA). Whiskers were made using Tukey’s method (whiskers extend to 1.5 × IQR). Ct values normalized to endogenous control *Gapdh* were compared with Welch’s *t*-test (2 tailed) and the FDR was corrected by Benjamini–Hochberg procedure. Normalized mean Ct values were compared using non-parametric Kruskal–Wallis H test (2-sided) followed by Dunn’s post hoc test. Significantly different inter-group differences in normalized mean Ct values expressed at a Bonferroni adjusted for multiple comparisons significance level of *P* < 0.05 indicated as < 0.05*, < 0.01**, and < 0.001***

The relative mRNA levels of 18 target genes tested in P-, NP^1^-, and NP^2^- HEI-OC1 cells are illustrated using mRNA copies of gene of interest per 1000 *Gapdh* mRNA (Fig. 6). Box and Whisker plots are presented for target genes’ Ct values normalized to *Gapdh* across these cultures (Figs. 7 and 8), and their *P* values (Kruskal–Wallis H test and Dunn’s post hoc, 2-sided) are summarized (Table 5). *Tubb1, Cdh1, Espn,* and *Sox2* mRNA levels were significantly elevated in non-permissive compared with permissive HEI-OC1 cells (*P* < 0.05, Bonferroni corrected Dunn’s test). *Atoh1*, *Tubb3, Myo7a, Pou4f3,* and *Zeb1* expressions were significantly reduced in P-HEI-OC1 cells when compared with NP^1^-HEI-OC1 cells (*P* < 0.01, Bonferroni corrected Dunn’s test). *Tubb5*, *Pax2*, *p27*^*Kip1*^*,* and *Vim* expressions were significantly decreased in NP^2^-HEI-OC1 cells (*P* < 0.01, Bonferroni corrected Dunn’s test) when compared with NP^1^- and/ or P- HEI-OC1 cells. *Krt18* expression was significantly increased (*P* < 0.01, Bonferroni corrected Dunn’s test) in NP^2^-HEI-OC1 cells when compared with P-HEI-OC1 cells. *Zeb2* expression level was comparable across all three cultures. *Sfmbt2* expression was comparable between P- and NP^1^- HEI-OC1 cells, whereas it was not detected in NP^2^-HEI-OC1 cells at Ct < 40.0. *Slc26a5* was relatively poorly expressed in all three cultures.

**Table 5:** Target gene expressions in P-, NP^1^- and NP^2^- HEI-OC1 cells

	Normalized mean Ct (± SD)			Kruskal–Wallis H test	NP^1^- vs. P-	NP^2^- vs. P-	NP^2^- vs. NP^1^-
Target genes	P-	NP^1^-	NP^2^-	*P*^*a*^ value	*P*^*a*^ value	*P*^*a*^ value	*P*^*a*^ value
*Atoh1*	15.12 (0.93)	6.85 (0.74)	7.25 (0.25)	0.013	0.005**	0.066	0.834
*Tubb1*	14.2 (0.26)	6.27 (0.62)	6.72 (0.98)	< 0.001	< 0.001***	0.003**	0.342
*Tubb3*	8.07 (0.15)	4.51 (0.54)	6.3 (1.03)	< 0.001	< 0.001***	0.036	0.056
*Tubb5*	-0.40 (0.39)	-0.91 (2.35)	4.58 (3.26)	< 0.001	0.423	0.002**	< 0.001**
*Cdh1*	13.6 (0.25)	5.91 (1.12)	7.4 (0.81)	< 0.001	< 0.001***	0.010*	0.086
*Espn*	12.81 (0.45)	8.37 (2.69)	9.19 (0.01)	0.004	0.002**	0.015*	0.642
*Myo7a*	13.07 (0.81)	8.13 (0.7)	12.25 (0.22)	0.001	< 0.001***	0.509	0.105
*Pou4f3*	14.61 (0.49)	6.68 (1.18)	7.3 (0.91)	0.003	0.001**	0.025	0.566
*Slc26a5*	n/d	n/d	n/d	n/a	n/a	n/a	n/a
*Sox2*	16.07 (0.59)	7.48 (1.54)	6.63 (0.32)	0.001	0.002**	0.001**	0.570
*p27*^*Kip1*^	5.98 (0.57)	1.91 (1.23)	8.46 (0.47)	0.007	0.117	0.117	0.002**
*Pax2*	5.42 (0.83)	2.84 (1.48)	10.8 (2.23)	< 0.001	0.029	0.104	< 0.001***
*Krt18*	18.47 (0.25)	14.97 (0.42)	13.06 (0.75)	0.022	0.167	0.007**	0.070
*Nes*	4.58 (0.42)	2.12 (0.99)	6.24 (1.21)	< 0.001	0.019	0.114	< 0.001***
*Sfmbt2*	9.73 (0.21)	8.87 (1.56)	n/d	0.099	n/a	n/a	n/a
*Vim*	-0.70 (1.4)	-2.47 (1.72)	3.26 (1.31)	< 0.001	0.070	0.007**	< 0.001***
*Zeb1*	7.02 (0.48)	5.11 (0.83)	6 (0.81)	0.001	< 0.001***	0.026	0.147
*Zeb2*	5.84 (0.37)	3.77 (1.85)	5.94 (0.4)	0.030	0.019	0.735	0.044

Normalized mean Ct values were compared using non-parametric Kruskal–Wallis H test (2-sided) followed by Dunn’s post hoc test. Significantly different inter-group differences in normalized mean Ct values expressed at a Bonferroni adjusted for multiple comparisons significance level of *P* < 0.05 indicated as < 0.05*; < 0.01**; < 0.001***

*CT* cycle threshold, *SD* standard deviation, actual *P* value (*P*^*a*^), not determined (n/d), not applicable (n/a)

**Fig. 9:**
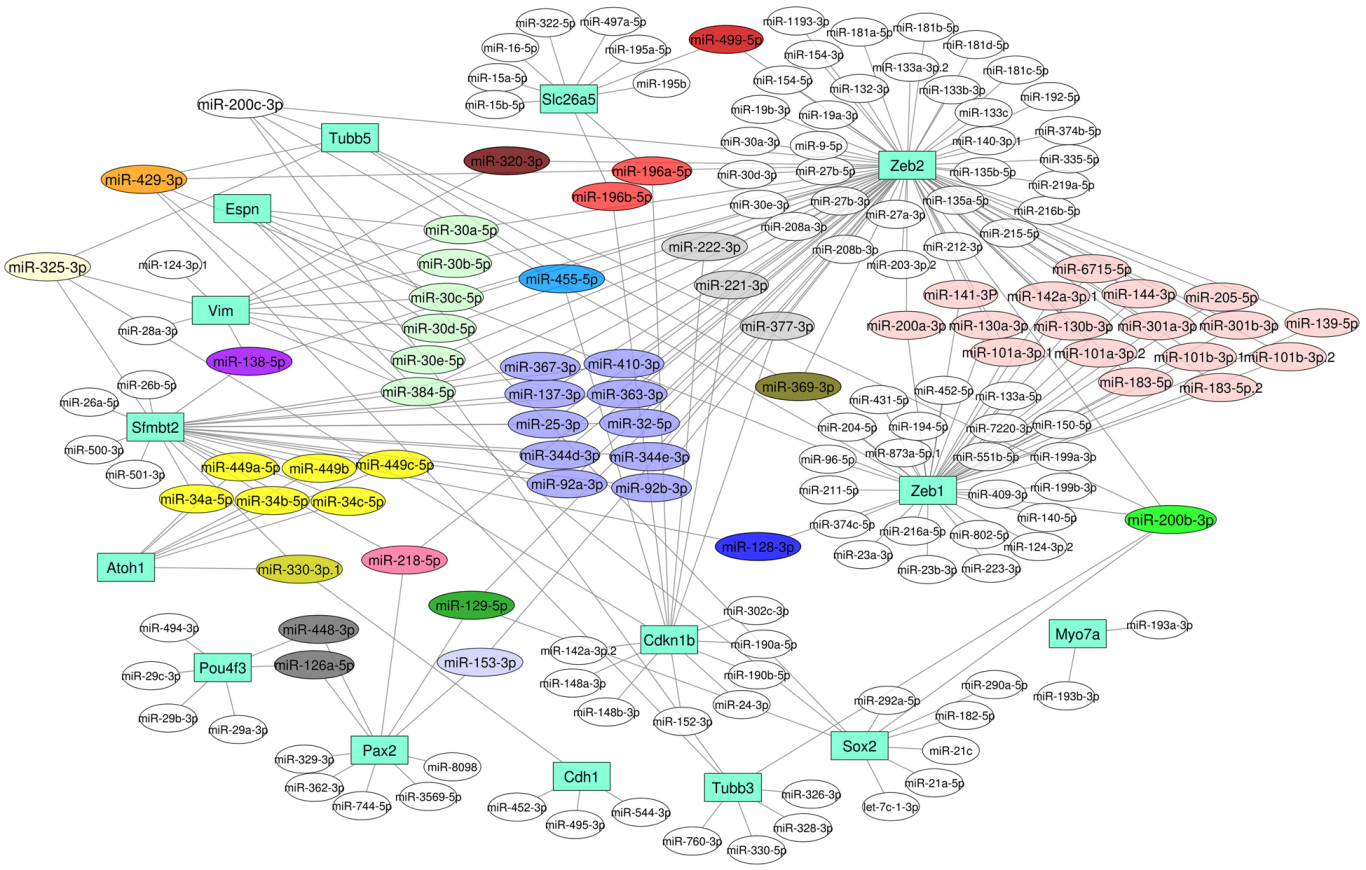
TargetScan predicted mRNA-miRNA interactions. Target genes and their biological targeting miRNAs that shared conserved (8mer and 7mer seed regions) sequences are summarized. *Atoh1* and *Sfmbt2* shared conserved sequences with miR-34a-5p/ -34b-5p/ -34c-5p/ -449a-5p, 449b and 449c-5p (yellow); *Sox2, Tubb3, Tubb5, Zeb1, and Zeb2* with miR-200c-3p (white) and miR-200b-3p with (green); *Tubb5, p27*^*Kip1*^* (Cdkn1b), Sfmbt2,* and *Vim* with miR-325-3p (butter); *Tubb3, Tubb5, Sox2, Zeb1,* and *Zeb2* with miR-429-3p (orange); *Espn, Vim,* and *Zeb2* with miR-30a-5p/ -30b-5p/ -30c-5p/-30d-5p/ -30e-5p and -384-5p (light green); prestin *(Slc26a5)* and *p27*^*Kip1*^ with miR-196a-5p and miR-196b-5p (light red); *Pax2, Sfmbt2,* and Z*eb2* with miR-218-5p (dark pink); *Pax2, Sox2,* and *Zeb2* with miR-129-5p (dark green); *Atoh1,* e-cadherin *(Cdh1),* and *Sfmbt2* with miR-330-3p.1 (goldenrod); *Tubb5*, Z*eb1* and Z*eb2* with miR-369-3p (olive); *Sfmbt2, Vim* and *Zeb2* with miR-138-5p (purple); *p27*^*Kip1*^ and *Zeb2* with miR-377-3p, -222-3p and -221-3p (light grey); *Pax2* and *Pou4f3* with miR-126a-5p and -448-3p (dark grey); *Sfmbt2* and *Zeb2* with miR-92a-3p/ -92b-3p/ -344d-3p/ -344e-3p/ -25-3p/ -32-5p/ -137-3p/ -363-3p/ -367-3p and -410-3p (light purple); Z*eb1* and *Zeb 2* with miR-200a-3p/ -183-5p/ -183-5p.2/ -101a-3p.1/ -101a-3p.2/ -101b-3p.1/ -101b-3p.2/ -130a-3p/ -130b-3p/ -301a-3p/ -301b-3p/ -139-5p/ -141-3p/ -142a.3p.1/ -144-3p/ -205-5p and -6715-5p (light pink); *Vim* and *Zeb2* with miR-320-3p (maroon); *Tubb5* and *p27*^*Kip1*^ with miR-455-5p (light blue); *Sfmbt2* and *Zeb1* with miR-128-3p (dark blue); *Pax2* and *Zeb2* with miR-153-3p (lavender); and *Slc26a5* and *Zeb2* with miR-499-5p (dark red) were shared conserved sequences. Source nodes were genes (turquoise, rectangle) and the target nodes were miRNAs (different colors, ellipse)

These target genes were primarily selected from the literature. Therefore, we searched their biological target miRNAs using TargetScanMouse version 7.2 (Fig. 9). Most interestingly, *Atoh1* and *Sfmbt2* showed conserved sites that match the seed regions of miR-34a-5p/ -34b-5p/ -34c-5p/ -449a-5p, 449b, and 449c-5p. Likewise, miR-200c-3p and -200b-3p target *Sox2, Tubb3, Tubb5, Zeb1,* and *Zeb2.* miR-30 family miRNAs -30a-5p/ -30b-5p/ 30c-5p/ -30d-5p and -30e-5p target *Espn, Vim,* and *Zeb2*. miR-196a-5p and -196b-5p target *Slc26a5* and *p27*^*Kip1*^* (Cdkn1b)*, miR-301a-3p and -301b-3p target *Zeb1* and *Zeb2*, and miR-222-3p targets *Zeb2* and *p27*^*Kip1*^. *Tubb1, Nes*, and *Krt18* did not show conserved sites that match the seed regions of any miRNAs.

### Fluorescence Immunocytochemistry (ICC) on HEI-OC1 Cells

**Fig. 10:**
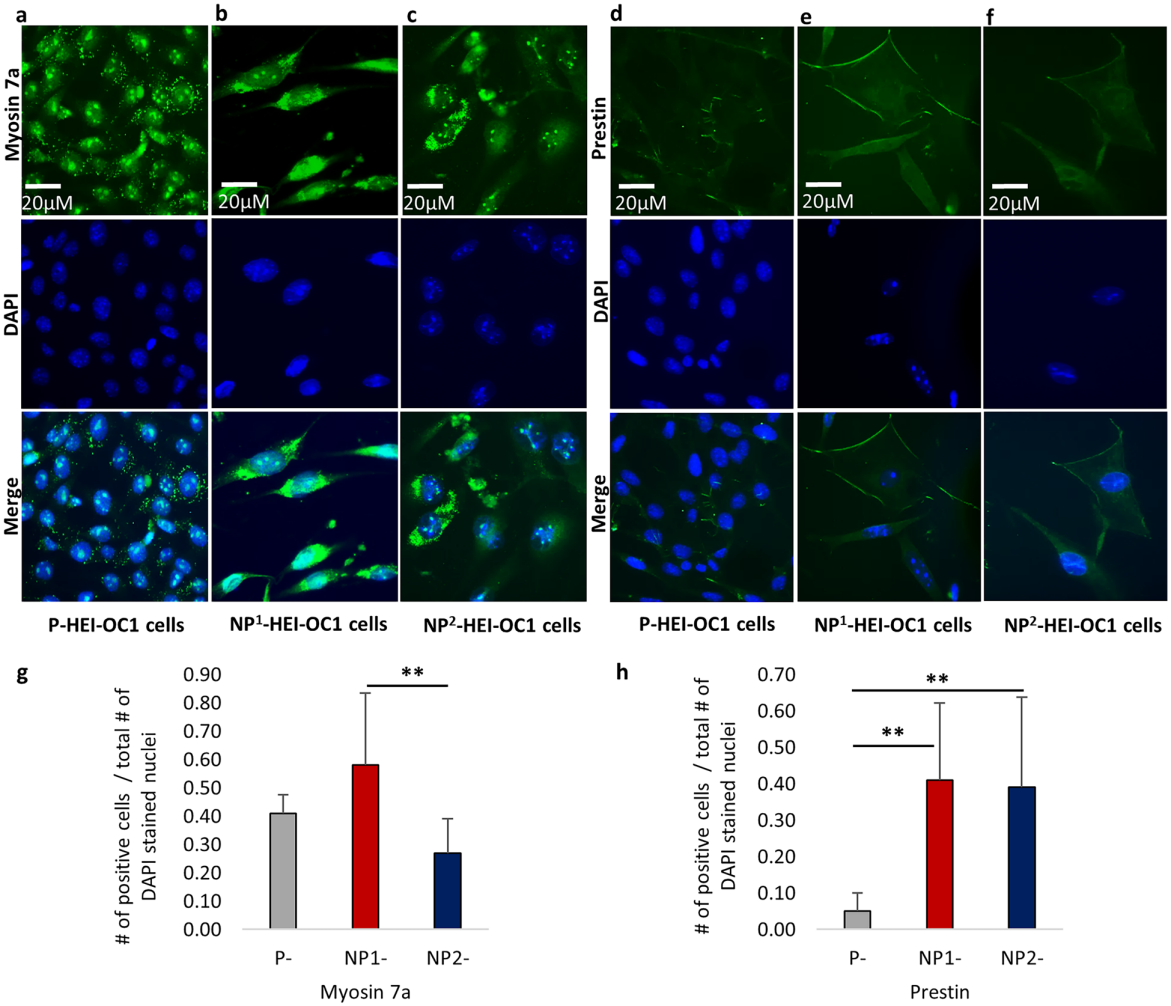
Immunofluorescence staining of inner ear hair cell markers in permissive and non-permissive HEI-OC1 cells. **a** Myosin 7a–positive apical projections, diffusely packed in P-HEI-OC1 cells. **b** and **c** Myosin 7a–positive apical projections, densely packed in NP^1^- and NP^2^-HEI-OC1 cells, respectively. **d** Relatively weak plasma membrane localization of prestin in P-HEI-OC1 cell. **e** Stable and strong prestin plasma membrane localization in NP^1^-HEI-OC1 cells. **f** Slightly unstable prestin expression in NP^2^-HEI-OC1 cells. **g** and **h** Proportion of myosin 7a– and prestin-positive cells, respectively in P-, NP^1^-, and NP^2^-HEI-OC1 cells (error bars indicate standard deviation). DAPI was used to stain the nuclei. Phase contrast microscopic images are presented with scale bar. Proportions of antibody-stained cells (positive) were compared using non-parametric Kruskal–Wallis *H* test (2-sided) followed by Dunn’s post hoc test. Significant inter-group differences in the proportion of positive cells expressed at a Bonferroni adjusted for multiple comparisons significance level of *P* < 0.05 indicated as < 0.05*, < 0.01**, and < 0.001***

**Fig. 11:**
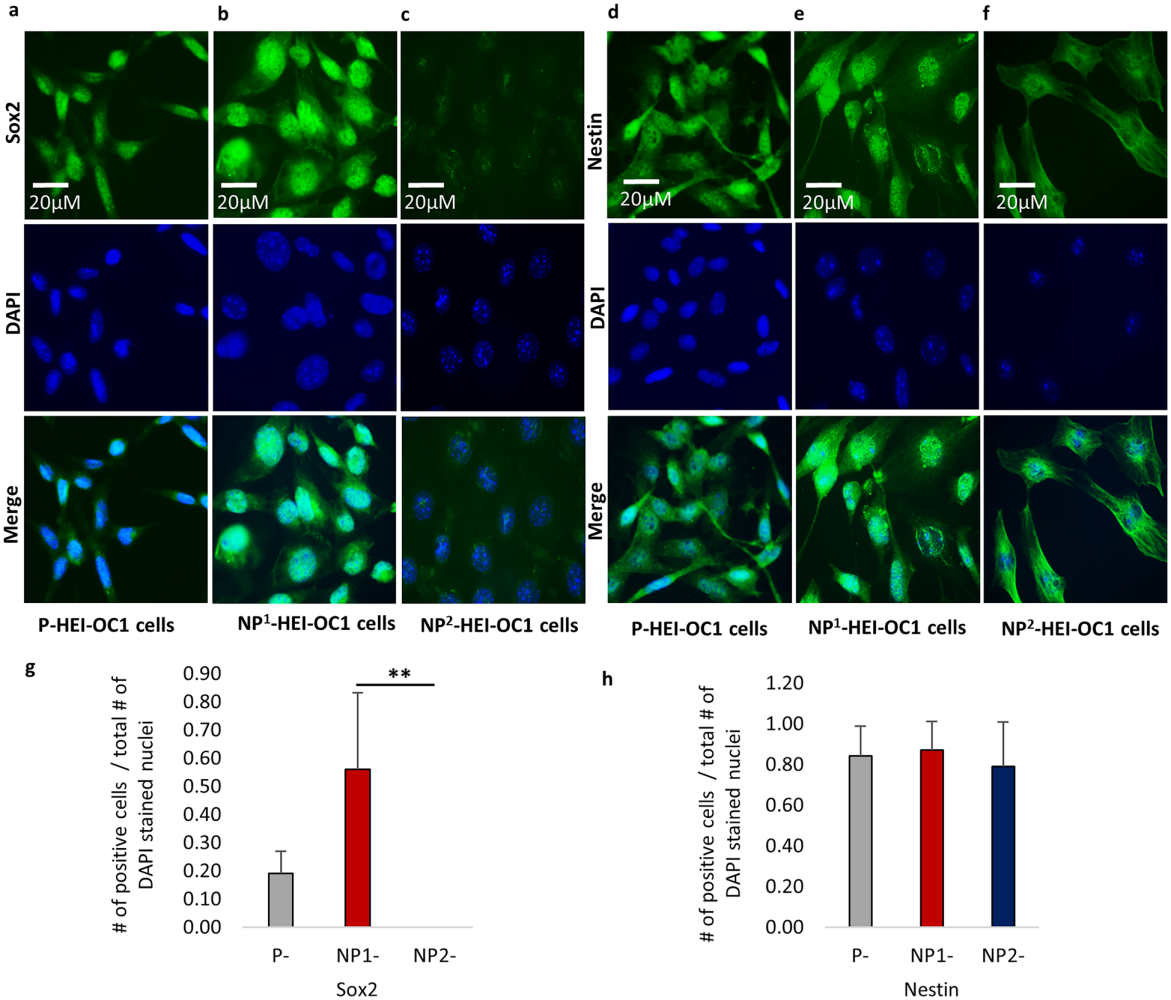
Immunofluorescence staining of stem/progenitor cell markers in permissive and non-permissive HEI-OC1 cells. **a** and **b** Sox2-positive nuclei identified in P- and NP^1^-HEI-OC1 cells, respectively. **c** Low/absence of Sox2-positive nuclei in NP^2^-HEI-OC1 cells. **d** and **e** Nestin-positive nuclei identified in P- and NP^1^-HEI-OC1 cells, respectively. **f** Nestin localized in the cytoplasm of differentiated NP^2^-HEI-OC1 cells. **g** and **h** Proportion of Sox2- and nestin-positive cells, respectively in P-, NP^1^-, and NP^2^-HEI-OC1 cells (error bars indicate standard deviation). DAPI was used to stain the nuclei. Phase contrast microscopic images are presented with scale bar. Proportions of antibody-stained cells (positive) were compared using non-parametric Kruskal–Wallis H test (2-sided) followed by Dunn’s post hoc test. Significant inter-group differences in the proportion of positive cells expressed at a Bonferroni adjusted for multiple comparisons significance level of *P* < 0.05 indicated as < 0.05*, < 0.01**, and < 0.001***

**Fig. 12:**
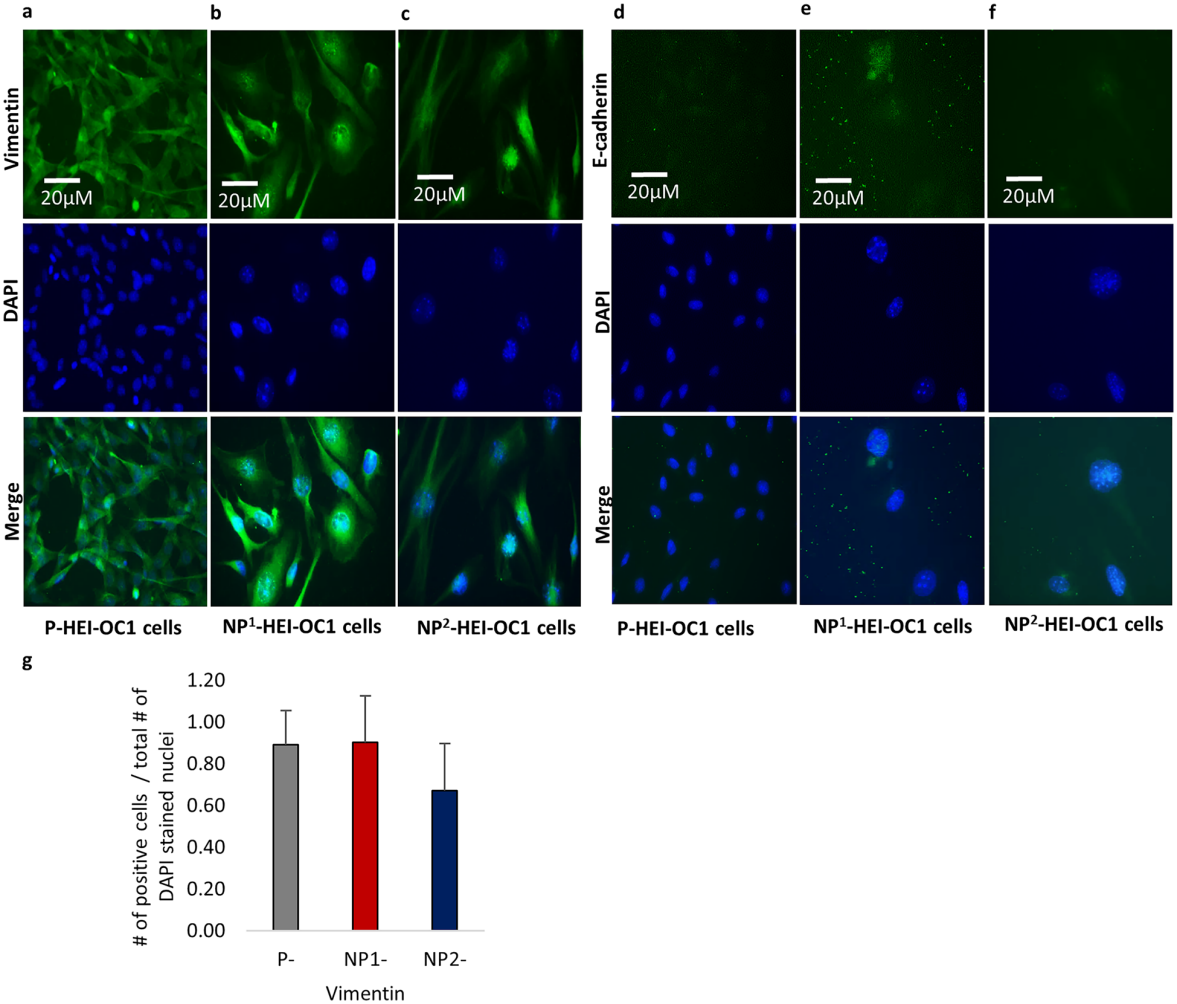
Immunofluorescence staining of EMT markers in permissive and non-permissive HEI-OC1 cells. **a, b,** and **c** Vimentin, a mesenchymal cell marker identified in P-, NP^1^-, and NP^2^-HEI-OC1 cells, respectively. **d, e,** and **f** Weak signals obtained for cell–cell adhesion marker e-cadherin in P-, NP^2^-, and NP^1^-HEI-OC1 cells, respectively. **g** Proportion of vimentin-positive cells in P-, NP^1^-, and NP^2^-HEI-OC1 cells, respectively (error bars indicate standard deviation). DAPI was used to stain the nuclei. Phase contrast microscopic images are presented with scale bar. Proportions of antibody-stained cells (positive) were compared using non-parametric Kruskal–Wallis H test (2-sided) followed by Dunn’s post hoc test. Significant inter-group differences in the proportion of positive cells expressed at a Bonferroni adjusted for multiple comparisons significance level of *P* < 0.05 indicated as < 0.05*, < 0.01**, and < 0.001***

Six protein markers myosin 7a, prestin, Sox2, nestin, e-cadherin, and vimentin were used to characterize HEI-OC1 cells maintained under permissive and non-permissive conditions, and the proportion of antibody-positive cells were analyzed using Kruskal–Wallis H test followed by Bonferroni-corrected Dunn’s post hoc test (Table 6) and presented for all three cultures (Figs. 10–12).

**Table 6:** Target protein expression in P-, NP^1^- and NP^2^- HEI-OC1 cells

	Mean (± SD)			Kruskal–Wallis H test	NP^1^- vs P-	NP^2^- vs. P-	NP^2^- vs. NP^1^-
Target proteins	P-	NP^1^-	NP^2^-	*P*^*a*^ value	*P*^*a*^ value	*P*^*a*^ value	*P*^*a*^ value
Myosin 7a	0.42 (0.06)	0.58 (0.25)	0.27 (0.12)	0.018	0.394	0.055	0.006**
Prestin	0.05 (0.05)	0.41 (0.21)	0.39 (0.24)	0.009	0.009**	0.007**	0.944
Sox2	0.19 (0.08)	0.56 (0.27)	0.00	0.002	0.130	0.052	0.001**
Nestin	0.84 (0.15)	0.86 (0.14)	0.79 (0.22)	0.960	n/a		
Vimentin	0.88 (0.16)	0.90 (0.22)	0.67 (0.23)	0.077	n/a		

Proportions of antibody-stained cells (positive) were compared using non-parametric Kruskal–Wallis H test (2-sided) followed by Dunn’s post hoc test. Significantly different inter-group differences in protein expressed at a Bonferroni adjusted for multiple comparisons significance level of *P* < 0.05 indicated as < 0.05*; < 0.01**; and < 0.001***

*SD* standard deviation, actual *P* value (*P*^*a*^), not applicable (n/a)

Myosin 7a–positive apical projections were dispersed in P-HEI-OC1 cells (Fig. 10a), whereas myosin 7a–positive apical projections were densely packed in NP^1^- and NP^2^- HEI-OC1 cells, respectively (Fig. 10b and c). Plasma membrane localization of prestin was identified in P-HEI-OC1 cells (Fig. 10d); however, signal was much stronger and stable in NP^1^-HEI-OC1 cells (Fig. 10e). Prestin expression was slightly unstable in NP^2^-HEI-OC1 cells (Fig. 10f) because of increasing cell’s vulnerability to multiple washing steps in the immunostaining procedures. The proportion of myosin 7a (*P* = 0.018, Kruskal–Wallis H test) and prestin (*P* = 0.009, Kruskal–Wallis H test)–positive cells differed significantly across P-, NP^1^-, and NP^2^- HEI-OC1 cell cultures (Table 6). The proportion of myosin 7a–positive cells was significantly reduced in NP^2^- compared to NP^1^-HEI-OC1 cells (*P* = 0.006, Bonferroni-corrected Dunn’s test) (Fig. 10g), whereas the proportion of prestin-positive cells was significantly decreased in P- compared with NP^1^- and NP^2^-HEI-OC1 cells (*P* = 0.009, *P* = 0.007, respectively, Bonferroni-corrected Dunn’s test) (Fig. 10h).

Sox2 expression was detected in both P- and NP^1^ -HEI-OC1 cells (Fig. 11a and b, respectively); on the other hand, Sox2 expression was diminished or disappeared in the nuclei of NP^2^-HEI-OC1 cells (Fig. 11c). NP^1^-HEI-OC1 cells contained both Sox2-positive and Sox2-negative cells. Sox2-negative cells could be considered as differentiated cells like Sox2-negative cells identified in NP^2^-HEI-OC1 cells. Between P- and NP^1^- HEI-OC1 cells, Sox2 was overexpressed or the signals were strong in NP^1^-HEI-OC1 cells, indicating its importance during hair cell differentiation. Nestin expression was strong in the nuclei of both P- and NP^1^- HEI-OC1 cells (Fig. 11d and e, respectively), whereas nestin expression was predominantly detected in the cytoplasm of differentiated NP^2^-HEI-OC1 cells (Fig. 11f). Sox2 expression was significantly decreased in NP^2^- compared with NP^1^- HEI-OC1 cell cultures (*P* = 0.001, Bonferroni-corrected Dunn’s test), whereas nestin expression was comparable across P-, NP^1^-, and NP^2^- HEI-OC1 cell cultures (*P* = 0.96, Kruskal–Wallis H test) (Table 6). The proportion of Sox2- and nestin-positive cells with respect to total number of DAPI-stained nuclei is presented (Fig. 11g and h).

Vimentin, a mesenchymal cell marker, was comparably expressed in all three HEI-OC1 cell cultures (Fig. 12a–c, respectively), and its semi quantification was not significantly different (*P* = 0.077, Kruskal–Wallis H test) among these cells (Fig. 12g). E-cadherin is an epithelial cell marker, involved in cell–cell adhesion. E-cadherin expression was comparatively weak in P-HEI-OC1 cell cultures (Fig. 12d). Due to increasing cell death and vulnerability to staining steps, remnants of e-cadherin protein were identified in non-permissive cells (Fig. 12e and f, respectively), and therefore, protein semi quantification was not carried out.

## Discussion

Inner ear tissue differentiation and maintenance are regulated and controlled by conserved sets of cell-specific miRNAs (Friedman et al. 2009). Here, we demonstrated differences in the miRNA signature of undifferentiated and differentiated HEI-OC1 cells.

Putative target genes of the DEMs in non-permissive HEI-OC1 cells revealed that MAPK, ErbB, and Ras signaling pathways were the predominantly significantly enriched KEGG pathways in differentiated HEI-OC1 cells (Fig. 3a and b). ErbBs are widely expressed in varying degrees in overlapping populations of sensory and non-sensory cells within the neonatal and adult inner ear (Hume et al. 2003). Hume et al. (2003) suggest that the expression of the ErbBs in supporting cells, hair cells, and non-sensory cells are potentially involved in the regulation of multiple processes including survival, synaptic maintenance, and cochlear homeostasis, in addition to a role in proliferation. In this current study, miRNA signature of non-permissive HEI-OC1 cells confirmed the functional enrichment of ErbB signaling pathway when compared with permissive HEI-OC1 cells. In addition to ErbB, Ras and MAPK signaling pathways were also enriched in non-permissive cells. Ras/MAPK pathway is essential in the regulation of cell cycle, differentiation, growth, and cell senescence, all of which are critical to normal growth and development (Tidyman and Rauen 2009). Haque et al. (2016), for the first time, showed that mitogen-activated protein 3 kinase 4 (MEKK4) signaling is highly regulated during inner ear development and is critical to normal cytoarchitecture and function as deficient mice exhibit a significant reduction of hair cells and hearing loss. Meanwhile, FoxO signaling was predominantly enriched in NP^2^-HEI-OC1 cells when compared with NP^1^-HEI-OC1 cells (Fig. 3c), suggesting the activation of cellular physiological events such as apoptosis with increasing incubation period.

DEMs miR-200-3p and miR-34c-3p were significantly and consistently upregulated in non-permissive cultures. The miR-200 family has been shown to inhibit epithelial to mesenchymal transition, by maintaining the epithelial phenotype through direct targeting of transcriptional repressors of e-cadherin (*Cdh1*), *Zeb1*, and *Zeb2* (Korpal and Kang 2008). *Cdh1* expression was significantly elevated in both NP^1^- and NP^2^- HEI-OC1 cells compared with P-HEI-OC1 cells (*P* ≤ 0.001 and *P* = 0.01 respectively, Bonferroni-corrected Dunn’s test) (Table 5), suggesting that upregulation of miR-200c-3p protects or maintains the epithelial characteristics in differentiated HEI-OC1 cells. Furthermore, *Vim* expression was significantly reduced in NP^2^- compared with P- and NP^1^- HEI-OC1 cells (*P* = 0.007 and *P* < 0.001, respectively, Bonferroni-corrected Dunn’s test) which is consistent with the *Cdh1* expression change and supports epithelialization in non-permissive HEI-OC1 cells. E-cadherin antibody staining in non-permissive cells also demonstrated (Fig. 12e and f). Cell culture morphology displayed the mesenchymal to epithelial transition when HEI-OC1 cells were transitioned from permissive to non-permissive conditions (Fig. 1d to b). Increased cell death in non-permissive cells and vulnerability to multiple washing steps likely contributed to the failure to detect e-cadherin protein. The expression level of *Zeb1* was significantly increased in NP^1^- compared with P-HEI-OC1 cells (*P* ≤ 0.001, Bonferroni-corrected Dunn’s test), whereas *Zeb2* expression was comparable across P-, NP^1^-, and NP^2^-HEI-OC1 cells (Table 5). Therefore, it is not possible to conclude that miR-200c-3p, exerts its effect on mesenchymal-to-epithelial transition in differentiated HEI-OC1 cells through transcriptional regulation of Z*eb1* or *Zeb2* (Fig. 9).

*Sox2* is also targeted by miR-200c-3p (Fig. 9). *Sox2*, an important transcription factor, plays multiple roles, most prominently in cellular reprogramming and stem cell pluripotency. In addition, *Sox2* is considered as a marker of the prosensory domain in the developing cochlea from which the cochlear and vestibular epithelia develop (Kiernan et al. 2005; Hume et al. 2007). Kempfle et al. (2016) reported that *Sox2* is required in the cochlea to both expand progenitor cells and initiate their differentiation into hair cells. This is supported by our study where fluorescence signals obtained for Sox2-positive cells were very strong in NP^1^- compared with P- HEI-OC1 cells (Fig. 11a and b). Furthermore, *Sox2* expression was significantly elevated in both NP^1^- and NP^2^- HEI-OC1 cells (*P* = 0.002 and *P* = 0.001, respectively, Bonferroni-corrected Dunn’s test) compared with P-HEI-OC1 cells (Table 5), whilst Sox2 protein expression was significantly (*P* = 0.001, Bonferroni-corrected Dunn’s test) decreased in NP^2^- compared with NP^1^-HEI-OC1 cells (Table 6). Low levels of Sox2 protein expression despite high levels of *Sox2* gene expression in the presence of miR-200c-3p upregulation in NP^2^-HEI-OC1 cells is consistent with miR-200c-3p’s inhibition of Sox2 protein synthesis by translational repression. It is likely that the effect of miR-200c-3p upregulation is sub-maximal in NP^1^-HEI-OC1 cell cultures which have not fully transitioned to a predominant differentiated cell culture as reflected by the presence of a mix of Sox2 antibody–positive and Sox2 antibody–negative cells.

Moreover, *Tubb3* and *Tubb5* are targets of miR-200c-3p (Fig. 9) based on their seed region homology. Microtubules are elaborated in a specific temporal pattern in the development of gerbil post-mitotic organ of Corti (Hallworth and Ludueña 2000; Hallworth et al. 2000). Jensen-Smith et al. (2003) describe that in the adult organ of Corti, each of the five major cells types synthesize a different subset of tubulin isotypes. To be specific, inner hair cells synthesize only Tubb1 and 2, while outer hair cells make Tubb1 and 4. Only Tubb2 and 4 are found in both inner and outer pillar cells, while Tubb1, 2, and 4 are present in Deiters cells, and Tubb1, 2, and 3 are found in organ of Corti dendrites. Tubb3 is commonly referred as the “neuron-specific” tubulin; however, Stone and Rubel (2000) reported Tubb3 in both mature and regenerating chick cochlea hair cells. We tested 3 tubulin isotypes: genes *Tubb1, 3,* and *5* in P-, NP^1^-, and NP^2^- HEI-OC1 cells. *Tubb1* expression was significantly elevated in both NP^1^- and NP^2^-HEI-OC1 cells (*P* < 0.001 and *P* = 0.003, respectively, Bonferroni-corrected Dunn’s test) compared with P- HEI-OC1 cells (Table 5), suggesting the presence of mature hair cells and/or organ of Corti dendrites in non-permissive cultures. *Tubb3* expression which is a mature neuron marker was elevated in NP^1^- but not NP^2^- when statistically compared with P-HEI-OC1 cells (Table 5). This is consistent with putative and validated genes targeted by DEMs in NP^1^- HEI-OC1 cells being predominantly overexpressed in biological processes: dendrite morphogenesis and nervous system development, respectively (Figs. 4a and 5b). *Tubb5* a target of miR-200c-3p a DEM in non-permissive cells was significantly reduced in NP^2^- compared with NP^1^- and P-HEI-OC1 cells (*P* = 0.002 and *P* < 0.001, respectively, Bonferroni-corrected Dunn’s test) (Table 5). Reduced level of *Tubb5* expression in NP^2^-HEI-OC1 cells suggests the inhibitory role of upregulated miR-200c-3p in differentiated HEI-OC1 cells possibly via mRNA degradation. In addition, *Espn*, which is required for the growth of hair cell stereocilia (Zheng et al. 2014), was significantly increased in NP^1^- and NP^2^- compared with P-HEI-OC1 cells (*P* = 0.002 and *P* = 0.015, respectively, Bonferroni-corrected Dunn’s test). miR-30 family miRNAs and miR-384-5p target *Espn* (Fig. 9). Though it is not statistically significant, miR-384-5p expression was downregulated (FC ≤ 0.1) in non-permissive cells (Suppl. Table 2).

The miR-34 (a/b/c) and miR-449 (a/b/c) families are two functionally related miRNA clusters (Bao et al. 2012; Lize et al. 2011, 2010). Simultaneous inactivation of miR-34b/c and miR-449 is reported to disrupt their target genes involved in cell fate control, brain development, and microtubule dynamics in mice (Wu et al. 2014). In our study, miR-34c-3p and -449a-5p, and miR-34c-3p miR-449b were significantly upregulated in NP^1^- and NP^2^- HEI-OC1 cells, respectively, when compared with P-HEI-OC1 cells (Tables 2 and 3). This observation suggests the possible role of upregulated miR-34 and miR-449 family members in microtubule dynamics and ciliogenesis of differentiated HEI-OC1 cells. However, a search of TargetScan failed to identify *Tubb1, Tubb3,* and *Tubb5* or *Espn* as targets of miR-34 and -449 family members (Fig. 9). On the other hand, miRNAs -34a-5p/ -34b-5p/ -34c-5p/ -449a-5p/ -449b, and 449c-5p were found to target *Atoh1* (Fig. 9)*. Atoh1* a proneural basic helix-loop-helix (bHLH) transcription factor that plays a major role in hair cell differentiation (Hongmiao et al. 2014) was significantly elevated in NP^1^-HEI-OC1 cells (*P* = 0.005, Bonferroni-corrected Dunn’s test) compared with P-HEI-OC1 cells (Table 5). The increased level of *Atoh1* expression with a parallel increase of miR-34c-3p/-449a-5p in NP^1^-HEI-OC1 cells (Table 1) is consistent with the miR-34/449 family’s role in promoting epithelial cell differentiation (Otto et al. 2017). However, the mechanism by which miR-34/ -449 family achieves this effect in HEI-OC1 cells needs further investigation*.*

Several of the miRNAs implicated in mouse 3′UTR evolution derive from a single rapidly expanded rodent-specific miRNA cluster located in the intron of *Sfmbt2*, a maternally imprinted polycomb gene. These miRNAs are expressed in both embryonic stem cells and the placenta (Zheng et al. 2011). miR-297 s, miR-466 s, miR-467 s, and miR-669 s fall into the *Sfmbt2* miRNA cluster in the 10^th^ intron of chromosome 2, based on sequence similarity (Zheng et al. 2011). miR-467a an abundant member of the *Sfmbt2* cluster promotes cell proliferation, and the remaining members of this cluster are enriched in pathways that regulate cellular growth (Zheng et al. 2011). In our study, *Sfmbt2* expression was found comparable between P- and NP^1^-HEI-OC1 cells, whereas it was not determined in NP^2^-HEI-OC1 cells (Fig. 8). Significantly downregulated miR-466a-3p in NP^1^-HEI-OC1 cells and miR-467a-5p in NP^2^-HEI-OC1 cells suggest the changes in cellular growth and lack of proliferation, respectively. As like *Atoh1*, *Sfmbt2* showed conserved sequences that match the seed regions of miR-34 and miR-449 family miRNAs. Therefore, we propose that the coordinated regulation of *Atoh1, Sfmbt2,* and miR-34/-449 family miRNAs could play a vital role in HEI-OC1 cell proliferation and differentiation. In addition, miR-17 family miRNAs -17-5p and -20a-3p and its paralogous -106a-5p were significantly downregulated in NP^2^-HEI-OC1 cells (Tables 3 and 4). Downregulation of these miRNAs has been reported to be associated with ageing and senescence (Hackl et al. 2010).

In addition, *Myo7a* which is expressed in the apical stereocilia as well as the cytoplasm of the inner and outer hair cells (Hasson et al. 1995) and *Pouf43* which has a central function in the development of all hair cells in human and mouse inner ear sensory epithelia (Hertzano et al. 2004) were significantly elevated in NP^1^- compared to P-HEI-OC1 cells (*P* < 0.001 and *P* = 0.001, respectively, Bonferroni-corrected Dunn’s test) (Table 5). *Pax2* which is one of the earliest genes in preotic tissue contributing to the inner ear development and governs the differentiation of precursor cells into various cell types (Christophorou et al. 2010) and *p27*^*Kip1*^ which provides a link between developmental control of cell proliferation and the morphological development of the inner ear (Chen and Segil 1999) were significantly reduced in NP^2^-HEI-OC1 cells (*P* < 0.001 and *P* = 0.002, respectively, Bonferroni-corrected Dunn’s test), suggesting the achievement of maturation when compared to NP^1^-HEI-OC1 cells (Table 5). Several miRNAs identified as potentially targeting *Myo7a*, *Pou4f3,* and *Pax2* (Fig. 9) were not sought in this study. *Nes* expression was significantly increased in NP^1^-HEI-OC1 cells (*P* < 0.001, Bonferroni-corrected Dunn’s test) compared with NP^2^-HEI-OC1 cells (Table 5) and its protein localized within the nucleus (Fig. 11e), suggesting the persistence of progenitor cell characteristics in this culture. Prestin (*Slc26a5*) and cytokeratin 18 (*Krt18*) expression were low in all three HEI-OC1 cell cultures (Fig. 6), in contrast to adult porcine derived inner ear cells where *Krt18* and *Slc26a5* gene expressions are highly expressed and positively correlated (Wijesinghe et al. 2021a, b). However, significantly elevated *Krt18* in NP^2^- compared with P-HEI-OC1 cells (*P* = 0.007, Bonferroni-corrected Dunn’s test) supported the presence of adult inner ear cell characteristics in fully differentiated HEI-OC1 cells. Likewise, prestin plasma membrane localization which reflects a differentiated outer hair cell characteristic (Park et al. 2016) was strong in non-permissive HEI-OC1 cells compared to permissive HEI-OC1 cells (Fig. 10d-f) reflecting a significantly higher level of prestin protein in both NP^1^- and NP^2^- HEI-OC1 cells (*P* = 0.009 and *P* = 0.007, respectively, Bonferroni-corrected Dunn’s test). It is notable that miR-196a-5p and miR-322-5p that target *Slc26a5* are differentially downregulated in NP^2^-HEI-OC1 cells (Table 3, Fig. 9) which is consistent with a reduction in miRNA inhibition of prestin gene function and can partly explain the relative abundance of prestin in differentiated HEI-OC1 cells. Downregulation of these miRNAs may not be measurably significant in NP^1^-HEI-OC1 cells which have not fully transitioned. Kalinec et al. (2016b) consider 2 weeks under non-permissive conditions the minimum time to achieve cultures of predominantly differentiated HEI-OC1 cells and that under that time there will still be a high level of undifferentiated cells. It is also important to note that miR-196a/b targets *Slc26a5* and *p27*^*Kip1*^ (Fig. 9) and their interactions in HEI-OC1 cells require further investigation.

There are some limitations to this study. The number of viable cells reduced considerably under non-permissive conditions, necessitating the adoption of a semi-quantitative immunofluorescence approach to determine the protein expression levels under the different culture conditions. In addition, the treated surfaces of the chamber slides (Lab Tek, Permanox TC Surface) used for fluorescence staining could have induced hair cell differentiation, resulting in inconsistencies between the gene and protein expression findings (Wijesinghe et al. 2021a, b; Liu et al. 2016). COVID-19 pandemic restrictions on lab access and reagents/laboratory supplies prevented PCR prestin optimization with multiple primer sets. In future work, we aim to explore the impact of the DEM changes on protein expressions under permissive and non-permissive culture conditions.

Despite these limitations, the distinct miRNA signature of differentiated HEI-OC1 cells could help in understanding miRNA-mediated cellular responses in the adult cochlea. Our findings suggest the potential mRNA-miRNA interactions that could be used in future inner ear hair cell regeneration and therapeutic studies.

## Supplementary Information

Below is the link to the electronic supplementary material.

Supplementary file1 (XLSX 63 KB)

## Data Availability

Data transparency will be maintained if required.

## References

[CR1] Agarwal V, Bell GW, Nam JW, Bartel DP (2015). Predicting effective microRNA target sites in mammalian mRNAs. Elife.

[CR2] Bao J, Li D, Wang L, Wu J, Hu Y, Wang Z, Chen Y, Cao X, Jiang C, Yan W, Xu C (2012). MicroRNA-449 and microRNA-34b/c function redundantly in murine testes by targeting E2F transcription factor-retinoblastoma protein (E2F-pRb) pathway. J Biol Chem.

[CR3] Benjamini Y, Hochberg Y (1995). Controlling the false discovery rate: a practical and powerful approach to multiple testing. Journal of the Royal Statistical Society B.

[CR4] Calin GA, Croce CM (2006). MicroRNA signatures in human cancers. Nat Rev Cancer.

[CR5] Chen P, Segil N (1999). p27(Kip1) links cell proliferation to morphogenesis in the developing organ of Corti. Development.

[CR6] Christophorou NA, Mende M, Lleras-Forero L, Grocott T, Streit A (2010). Pax2 coordinates epithelial morphogenesis and cell fate in the inner ear. Dev Biol.

[CR7] Cyr JL, Bell AM, Hudspeth AJ (2000). Identification with a recombinant antibody of an inner-ear cytokeratin, a marker for hair-cell differentiation. Proc Natl Acad Sci U S A.

[CR8] Devarajan P, Savoca M, Castaneda MP, Park MS, Esteban-Cruciani N, Kalinec G, Kalinec F (2002). Cisplatin-induced apoptosis in auditory cells: role of death receptor and mitochondrial pathways. Hear Res.

[CR9] Esquela-Kerscher A, Slack FJ (2006). Oncomirs - microRNAs with a role in cancer. Nat Rev Cancer.

[CR10] Filipowicz W, Bhattacharyya SN, Sonenberg N (2008). Mechanisms of post-transcriptional regulation by microRNAs: are the answers in sight?. Nat Rev Genet.

[CR11] Friedman LM, Dror AA, Mor E, Tenne T, Toren G, Satoh T, Biesemeier DJ, Shomron N, Fekete DM, Hornstein E, Avraham KB (2009). MicroRNAs are essential for development and function of inner ear hair cells in vertebrates. Proc Natl Acad Sci U S A.

[CR12] Hackl M, Brunner S, Fortschegger K, Schreiner C, Micutkova L, Mück C, Laschober GT, Lepperdinger G, Sampson N, Berger P, Herndler-Brandstetter D, Wieser M, Kühnel H, Strasser A, Rinnerthaler M, Breitenbach M, Mildner M, Eckhart L, Tschachler E, Trost A, Bauer JW, Papak C, Trajanoski Z, Scheideler M, Grillari-Voglauer R, Grubeck-Loebenstein B, Jansen-Dürr P, Grillari J (2010). miR-17, miR-19b, miR-20a, and miR-106a are down-regulated in human aging. Aging Cell.

[CR13] Haque K, Pandey AK, Zheng HW, Riazuddin S, Sha SH, Puligilla C (2016). MEKK4 Signaling Regulates Sensory Cell Development and Function in the Mouse Inner Ear. J Neurosci.

[CR14] Hallworth R, Ludueña RF (2000). Differential expression of beta tubulin isotypes in the adult gerbil cochlea. Hear Res.

[CR15] Hallworth R, McCoy M, Polan-Curtain J (2000). Tubulin expression in the developing and adult gerbil organ of Corti. Hear Res.

[CR16] Hasson T, Heintzelman MB, Santos-Sacchi J, Corey DP, Mooseker MS (1995). Expression in cochlea and retina of myosin VIIa, the gene product defective in Usher syndrome type 1B. Proc Natl Acad Sci U S A.

[CR17] Hasson T, Gillespie PG, Garcia JA, MacDonald RB, Zhao Y, Yee AG, Mooseker MS, Corey DP (1997). Unconventional myosins in inner-ear sensory epithelia. J Cell Biol.

[CR18] Hertzano R, Montcouquiol M, Rashi-Elkeles S, Elkon R, Yücel R, Frankel WN, Rechavi G, Möröy T, Friedman TB, Kelley MW, Avraham KB (2004). Transcription profiling of inner ears from Pou4f3(ddl/ddl) identifies Gfi1 as a target of the Pou4f3 deafness gene. Hum Mol Genet.

[CR19] Hongmiao R, Wei L, Bing H, Xiong DD, Jihao R (2014). Atoh1: landscape for inner ear cell regeneration. Curr Gene Ther.

[CR20] da Huang W, Sherman BT, Lempicki RA (2009). Bioinformatics enrichment tools: paths toward the comprehensive functional analysis of large gene lists. Nucleic Acids Res.

[CR21] da Huang W, Sherman BT, Lempicki RA (2009). Systematic and integrative analysis of large gene lists using DAVID bioinformatics resources. Nat Protoc.

[CR22] Huang Y, Litvinov IV, Wang Y, Su MW, Tu P, Jiang X, Kupper TS, Dutz JP, Sasseville D, Zhou Y (2014). Thymocyte selection-associated high mobility group box gene (TOX) is aberrantly over-expressed in mycosis fungoides and correlates with poor prognosis. Oncotarget.

[CR23] Hume CR, Kirkegaard M, Oesterle EC (2003). ErbB expression: the mouse inner ear and maturation of the mitogenic response to heregulin. J Assoc Res Otolaryngol.

[CR24] Hume CR, Bratt DL, Oesterle EC (2007). Expression of LHX3 and SOX2 during mouse inner ear development. Gene Expr Patterns.

[CR25] Jat PS, Noble MD, Ataliotis P, Tanaka Y, Yannoutsos N, Larsen L, Kioussis D (1991). Direct derivation of conditionally immortal cell lines from an H-2Kb-tsA58 transgenic mouse. Proc Natl Acad Sci U S A.

[CR26] Jensen-Smith HC, Eley J, Steyger PS, Ludueña RF, Hallworth R (2003). Cell type-specific reduction of beta tubulin isotypes synthesized in the developing gerbil organ of Corti. J Neurocytol.

[CR27] Kalinec GM, Webster P, Lim DJ, Kalinec F (2003). A cochlear cell line as an in vitro system for drug ototoxicity screening. Audiol Neurootol.

[CR28] Kalinec G, Thein P, Park C, Kalinec F (2016). HEI-OC1 cells as a model for investigating drug cytotoxicity. Hear Res.

[CR29] Kalinec GM, Park C, Thein P, Kalinec F (2016). Working with Auditory HEI-OC1 Cells. J vis Exp.

[CR30] Kempfle JS, Turban JL, Edge AS (2016). Sox2 in the differentiation of cochlear progenitor cells. Sci Rep.

[CR31] Kiernan AE, Pelling AL, Leung KK, Tang AS, Bell DM, Tease C, Lovell-Badge R, Steel KP, Cheah KS (2005). Sox2 is required for sensory organ development in the mammalian inner ear. Nature.

[CR32] Kim VN, Han J, Siomi MC (2009). Biogenesis of small RNAs in animals. Nat Rev Mol Cell Biol.

[CR33] Korpal M, Kang Y (2008). The emerging role of miR-200 family of microRNAs in epithelial-mesenchymal transition and cancer metastasis. RNA Biol.

[CR34] Lewis MA, Quint E, Glazier AM, Fuchs H, De Angelis MH, Langford C, van Dongen S, Abreu-Goodger C, Piipari M, Redshaw N, Dalmay T, Moreno-Pelayo MA, Enright AJ, Steel KP (2009). An ENU-induced mutation of miR-96 associated with progressive hearing loss in mice. Nat Genet.

[CR35] Lewis MA, Buniello A, Hilton JM, Zhu F, Zhang WI, Evans S, van Dongen S, Enright AJ, Steel KP (2016). Exploring regulatory networks of miR-96 in the developing inner ear. Sci Rep.

[CR36] Li H, Kloosterman W, Fekete DM (2010). MicroRNA-183 family members regulate sensorineural fates in the inner ear. J Neurosci.

[CR37] Liu Q, Shen Y, Chen J, Ding J, Tang Z, Zhang C, Chen J, Li L, Chen P, Wang J (2016). Induction of functional hair-cell-like cells from mouse cochlear multipotent cells. Stem Cells Int.

[CR38] Lizé M, Klimke A, Dobbelstein M (2011). MicroRNA-449 in cell fate determination. Cell Cycle.

[CR39] Lizé M, Pilarski S, Dobbelstein M (2010). E2F1-inducible microRNA 449a/b suppresses cell proliferation and promotes apoptosis. Cell Death Differ.

[CR40] Lou X, Dong Y, Xie J, Wang X, Yang L, Tokuda M, Zhang Y (2014). Comparing the cultivated cochlear cells derived from neonatal and adult mouse. J Transl Med.

[CR41] Mahmoudian-Sani MR, Mehri-Ghahfarrokhi A, Ahmadinejad F, Hashemzadeh-Chaleshtori M, Saidijam M, Jami MS (2017). MicroRNAs: effective elements in ear-related diseases and hearing loss. Eur Arch Otorhinolaryngol.

[CR42] McDonald JH (2014) Handbook of Biological Statistics (3rd ed.). Sparky House Publishing, Baltimore, Maryland, 254–260

[CR43] Mencía A, Modamio-Høybjør S, Redshaw N, Morín M, Mayo-Merino F, Olavarrieta L, Aguirre LA, del Castillo I, Steel KP, Dalmay T, Moreno F, Moreno-Pelayo MA (2009). Mutations in the seed region of human miR-96 are responsible for nonsyndromic progressive hearing loss. Nat Genet.

[CR44] Nunez DA, Wijesinghe P, Nabi S, Yeh D, Garnis C (2020). microRNAs in sudden hearing loss. Laryngoscope.

[CR45] Obernosterer G, Leuschner PJ, Alenius M, Martinez J (2006). Post-transcriptional regulation of microRNA expression. RNA.

[CR46] Otto T, Candido SV, Pilarz MS, Sicinska E, Bronson RT, Bowden M, Lachowicz IA, Mulry K, Fassl A, Han RC, Jecrois ES, Sicinski P (2017). Cell cycle-targeting microRNAs promote differentiation by enforcing cell-cycle exit. Proc Natl Acad Sci U S A.

[CR47] Park C, Thein P, Kalinec G, Kalinec F (2016). HEI-OC1 cells as a model for investigating prestin function. Hear Res.

[CR48] Pasquinelli AE (2012). MicroRNAs and their targets: recognition, regulation and an emerging reciprocal relationship. Nat Rev Genet.

[CR49] Peláez N, Carthew RW (2012). Biological robustness and the role of microRNAs: a network perspective. Curr Top Dev Biol.

[CR50] Rudnicki A, Avraham KB (2012). microRNAs: the art of silencing in the ear. EMBO Mol Med.

[CR51] Sacheli R, Nguyen L, Borgs L, Vandenbosch R, Bodson M, Lefebvre P, Malgrange B (2009). Expression patterns of miR-96, miR-182 and miR-183 in the development inner ear. Gene Expr Patterns.

[CR52] Schmittgen TD, Livak KJ (2008). Analyzing real-time PCR data by the comparative C(T) method. Nat Protoc.

[CR53] Shannon P, Markiel A, Ozier O, Baliga NS, Wang JT, Ramage D, Amin N, Schwikowski B, Ideker T (2003). Cytoscape: a software environment for integrated models of biomolecular interaction networks. Genome Res.

[CR54] Soldà G, Robusto M, Primignani P, Castorina P, Benzoni E, Cesarani A, Ambrosetti U, Asselta R, Duga S (2012). A novel mutation within the MIR96 gene causes non-syndromic inherited hearing loss in an Italian family by altering pre-miRNA processing. Hum Mol Genet.

[CR55] Sticht C, De La Torre C, Parveen A, Gretz N (2018). miRWalk: An online resource for prediction of microRNA binding sites. PLoS ONE.

[CR56] Stone JS, Rubel EW (2000). Temporal, spatial, and morphologic features of hair cell regeneration in the avian basilar papilla. J Comp Neurol.

[CR57] Tidyman WE, Rauen KA (2009). The RASopathies: developmental syndromes of Ras/MAPK pathway dysregulation. Curr Opin Genet Dev.

[CR58] Untergasser A, Nijveen H, Rao X, Bisseling T, Geurts R, Leunissen JA (2007). Primer3Plus, an enhanced web interface to Primer3. Nucleic Acids Res.

[CR59] van Rooij E, Sutherland LB, Liu N, Williams AH, McAnally J, Gerard RD, Richardson JA, Olson EN (2006). A signature pattern of stress-responsive microRNAs that can evoke cardiac hypertrophy and heart failure. Proc Natl Acad Sci U S A.

[CR60] Wang Z, Liu Y, Han N, Chen X, Yu W, Zhang W, Zou F (2010). Profiles of oxidative stress-related microRNA and mRNA expression in auditory cells. Brain Res.

[CR61] Watanabe R, Morell MH, Miller JM, Kanicki AC, O'Shea KS, Altschuler RA, Raphael Y (2012). Nestin-expressing cells in the developing, mature and noise-exposed cochlear epithelium. Mol Cell Neurosci.

[CR62] Weston MD, Pierce ML, Rocha-Sanchez S, Beisel KW, Soukup GA (2006). MicroRNA gene expression in the mouse inner ear. Brain Res.

[CR63] Wijesinghe P, Nunez DA, Garnis C (2021a) Mirnas profiling of differentiated and undifferentiated HEI-OC1 cells: will it have an impact on auditory cell studies? Association For Research In Otolaryngology (ARO) 44th Annual Mid-Winter Meeting, Renaissance Sea World, Orlando Florida, USA, Feb 20^th^ to 24^th^

[CR64] Wijesinghe P, Sastry A, Hui E et al (2021b) Adult porcine (Sus scrofa) derived inner ear cells possessing multipotent stem/progenitor cell characteristics in in vitro cultures. bioRxiv. 10.1101/2021b.01.22.427339

[CR65] Wu J, Bao J, Kim M, Yuan S, Tang C, Zheng H, Mastick GS, Xu C, Yan W (2014). Two miRNA clusters, miR-34b/c and miR-449, are essential for normal brain development, motile ciliogenesis, and spermatogenesis. Proc Natl Acad Sci U S A.

[CR66] Yamasoba T, Kondo K (2006). Supporting cell proliferation after hair cell injury in mature guinea pig cochlea in vivo. Cell Tissue Res.

[CR67] Zheng L, Beeler DM, Bartles JR (2014). Characterization and regulation of an additional actin-filament-binding site in large isoforms of the stereocilia actin-bundling protein espin. J Cell Sci.

[CR68] Zheng GX, Ravi A, Gould GM, Burge CB, Sharp PA (2011). Genome-wide impact of a recently expanded microRNA cluster in mouse. Proc Natl Acad Sci U S A.

